# Safety and efficacy of intratumoural anti-CTLA4 with intravenous anti-PD1

**DOI:** 10.1038/s41586-026-10341-w

**Published:** 2026-04-29

**Authors:** Lambros Tselikas, Sandrine Susini, Matthieu Texier, Andrey Yurchenko, Emilie Routier, Mona Amini-Adle, Edi Tihic, Séverine Mouraud, François-Xavier Danlos, Samy Ammari, Thibault Raoult, Séverine Roy, Delphine Bredel, Siham Farhane, Lydie Cassard, Irma Molinaro, Alexander Eggermont, Jean-Charles Soria, Laurence Zitvogel, Christophe Massard, Angelo Paci, Thierry de Baere, Jean-Yves Scoazec, Nathalie Chaput, Sergey Nikolaev, Nicolas Meyer, Céleste Lebbé, Stéphane Dalle, Caroline Robert, Aurélien Marabelle

**Affiliations:** 1 INSERM CIC 1428, BIOTHERIS, Villejuif, France; 2 INSERM U1015, Laboratoire de Recherche Translationnelle en Immunothérapie (LRTI), Villejuif, France; 3 Gustave Roussy, Radiologie Interventionnelle, Département d’Anesthésie Chirurgie et Interventionnel (DACI), Villejuif, France; 4 Université Paris Saclay, Faculty of Medicine, Villejuif, France; 5 Gustave Roussy, Service de Biostatistiques et d’Epidémiologie (SBE), Université Paris Saclay, Villejuif, France; 6 INSERM U1018, ONCOSTAT, Equipe Labellisée Ligue contre le Cancer, Villejuif, France; 7 INSERM U981, Gustave Roussy, Villejuif, France; 8 Gustave Roussy, Dermatologie, Département de Médecine Oncologique, Villejuif, France; 9 Hospices Civils de Lyon, Département de Dermatologie, Lyon, France; 10 Gustave Roussy, Département d’Imagerie Médicale, Villejuif, France; 11 Gustave Roussy, Service de Promotion d’Etudes Cliniques, DRC, Villejuif, France; 12 Université Paris-Saclay, Gustave Roussy, INSERM, Laboratoire d’Immunomonitoring en Oncologie US23, Biothérapies Innovantes U1363, Villejuif, F-94805 France; 13 Gustave Roussy, Département de Biologie et Pathologie Médicale, Villejuif, France; 14 Gustave Roussy, Département d’Innovation Thérapeutique et des Essais Précoces, Villejuif, France; 15 INSERM U1015, Immunologie des tumeurs et immunothérapie contre le cancer, Villejuif, France; 16 Gustave Roussy, Service de Pharmacologie, Département de Biologie et Pathologie médicales, Villejuif, France; 17 CHU de Toulouse, Service d’Oncodermatologie, IUCT-O, Toulouse, France; 18 INSERM UMR 1037, Cancer Research Center of Toulouse (CRCT), Toulouse, France; 19 Université Toulouse III - Paul Sabatier, Département de Dermatologie, Toulouse, France; 20 Université Paris Cité, AP-HP Dermato-oncologie et CIC, Institut du Cancer APHP nord, Paris, France; 21 INSERM U1342–Equipe 1–CNRS EMR8000, Hôpital Saint Louis, Paris, France; 22 INSERM U1052-CNRS UMR5286, Plasticité Tumorale dans le Mélanome, Centre de Recherche en Cancérologie de Lyon, Centre Léon Bérard, Lyon, France; 23 Université Claude Bernard Lyon 1, Lyon, France

**Keywords:** Drug development, Immunoediting, Translational research, Melanoma

## Abstract

Intravenous administration of anti-CTLA4 with anti-PD1 provides durable tumour responses but causes severe treatment-related adverse events in patients with cancer^1^. Intratumoural administration at lower doses but high local concentrations could enhance antitumour efficacy while minimizing systemic exposure and toxicity. Here we report the randomized multicentre phase 1b NIVIPIT trial (ClinicalTrials.gov: NCT02857569), which enrolled 61 patients with untreated metastatic melanoma, randomly assigned 2:1 to receive intravenous nivolumab (anti-PD1; 1 mg kg^−1^) combined with either intratumoural ipilimumab (anti-CTLA4; 0.3 mg kg^−1^) or intravenous ipilimumab (3 mg kg^−1^). The primary end-point was met with significantly lower incidence of grade 3 or 4 treatment-related adverse events at 6 months in the intratumoural versus intravenous arm (22.6% versus 57.1%), equivalent to anti-PD1 monotherapy. RECIST (response evaluation criteria in solid tumours) best objective response rate reached 65.7% for anti-CTLA4 injected lesions and 50% for uninjected lesions, confirming the relationship between intratumoural exposure to anti-CTLA4 and efficacy. Baseline tumour immune profiling revealed that protumoural activated regulatory T (T_reg_) cells and M2 macrophages predict durable clinical benefit, regardless of the anti-CTLA4 administration route. A decrease in activated intratumoural T_reg_ cells occurred only in patients who showed durable clinical benefit, who also presented high intratumoural Fcγ receptor (FcγR) expression. Our results provide a rationale for intratumoural anti-CTLA4 strategies in oligometastatic and early-stage cancers and indicate that high intratumoural activated T_reg_ cell and FcγR^+^ M2 macrophage numbers are prerequisites for efficacy of combined anti-CTLA4 and anti-PD1.

## Abstract (web summary)

The phase 1b NIVIPIT trial shows that in combined therapy with intravenous nivolumab (anti-PD1), intratumoural administration of ipilimumab (anti-CTLA4) offers improved safety and greater efficacy compared with intravenous delivery.

## Main

Over the past decade, outcomes for metastatic melanoma have markedly improved, reaching 52% melanoma-specific overall survival at 10 years, driven largely by intravenous immunotherapies targeting the T cell co-inhibitory checkpoints CTLA4 and PD1^2^. Following the approval of ipilimumab (anti-CTLA4 immunoglobulin G1 (IgG1)) in 2011^3^ and nivolumab (anti-PD1 IgG4) in 2014^4,5^, combined nivolumab and ipilimumab therapy was approved in 2015 and incorporated into first line treatment guidelines^6^. Although this combination achieves higher response rates and survival than monotherapy^1^, it is associated with severe (common terminology criteria for adverse events (CTCAE) grade ≥3) treatment-related adverse events in approximately 60% of patients, some of which are irreversible and 1–4% of which are fatal in real-world settings^7,8^, limiting its use to about half of eligible patients in routine clinical practice^9^. Approved anti-PD1 monoclonal antibodies act as checkpoint blockers through their non-depleting IgG4 isotype. Once PD1 receptor saturation is achieved (approximately 0.3 mg kg^−1^), dose escalation does not improve efficacy or meaningfully increase toxicity^10,11^. By contrast, ipilimumab is an unmodified IgG1 antibody that is capable of engaging Fcγ receptor (FcγR) proteins and inducing antibody-dependent cellular cytotoxicity and phagocytosis^12^, resulting in dose-dependent increases in both efficacy and toxicity^13^.

Preclinical studies indicate that intratumoural delivery of low doses of anti-CTLA4 at high local concentrations can reduce systemic exposure while preserving dose-dependent antitumour activity^14–17^. A phase 1 study in patients with stage III–IV melanoma (*n* = 12) evaluating intratumoural ipilimumab combined with intratumoural interleukin-2 showed promising activity without dose-limiting toxicity, with objective responses in 67% of injected lesions and abscopal responses in 40% of evaluable patients^18^.

Based on these data, we hypothesized that administering a tenfold lower total dose of ipilimumab (0.3 mg kg^−1^) directly into tumours at the highest commercially available concentration (5 mg ml^−1^) could enhance antitumour efficacy while limiting systemic toxicity. To address this hypothesis, we initiated a multicentre randomized phase 1b trial evaluating the safety, efficacy and associated biomarkers of intratumoural ipilimumab combined with intravenous nivolumab in patients with previously untreated advanced melanoma.

**Extended Data Fig. 1:**
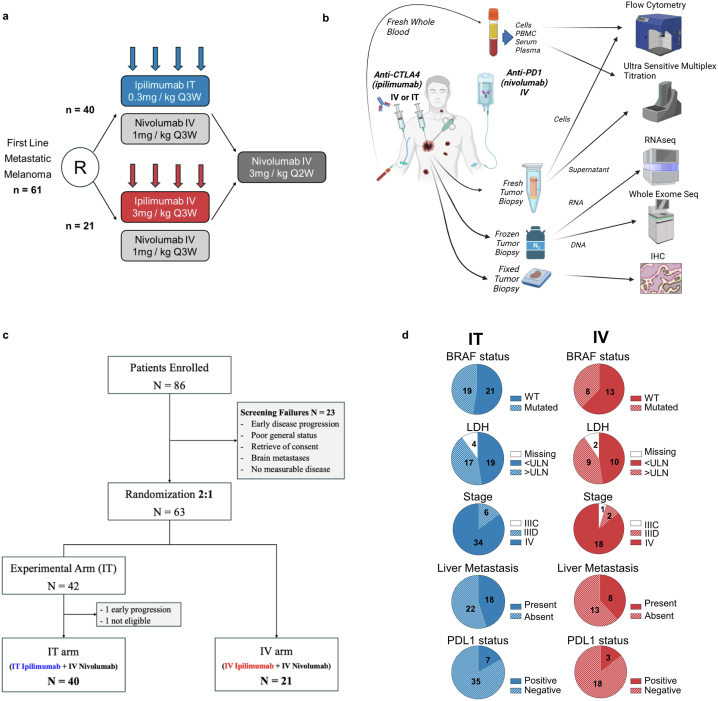
NIVIPIT Trial Design and Characteristics of the Randomized Patients. **a**. Clinical trial 2:1 randomization design between the ipilimumab intratumoral (IT) and intravenous (IV) treatment arms. **b**. Patient sampling and pre-planned prospective biomarker explorations **c**. CONSORT diagram of the patients enrolled and randomized **d**. Clinical and biological prognostic factors of the patients enrolled in the ipilimumab IT or IV arms of the trial.

The NIVIPIT trial (ClinicalTrials.gov: NCT02857569) evaluated intravenous nivolumab (1 mg kg^−1^) combined with either intravenous ipilimumab at 3 mg kg^−1^ (IV arm) or intratumoural ipilimumab at 0.3 mg kg^−1^ (IT arm), administered every 3 weeks for 4 doses, followed by nivolumab 3 mg kg^−1^ every 2 weeks for up to 12 months (Extended Data Fig. 1a). A pharmacokinetic, pharmacodynamic and biomarker programme including prospective analyses of fresh tumour biopsies and blood samples was incorporated into the protocol (Extended Data Fig. 1b).

Sixty-one patients were enrolled across 4 clinical sites and randomized in a 2:1 ratio to the IT arm (*n* = 40) or the IV arm (*n* = 21) (Methods, Extended Data Fig. 1c and Extended Data Table 1). Randomization was stratified by metastatic stage, tumour BRAF status and tumour PD-L1 expression, resulting in well-balanced treatment arms regarding major prognostic factors, including liver metastases and lactate dehydrogenase levels (Extended Data Fig. 1d).

A total of 162 intratumoural injections were performed across 46 tumour lesions, most frequently lymph node and cutaneous or subcutaneous metastases, although liver and lung lesions were also injected. The mean and median baseline diameters of injected lesions were 32.7 mm and 26 mm, respectively (s.d. = 23.0).

## Safety profile of intratumoural anti-CTLA4

**Fig. 1:**
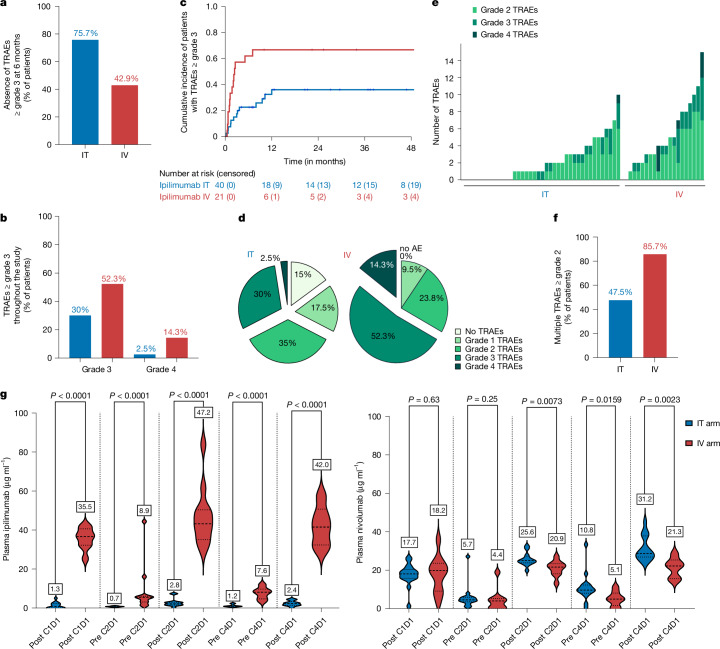
Safety of intratumoural ipilimumab in combination with intravenous nivolumab. **a**, Proportion of patients without grade 3 or 4 treatment-related adverse events (TRAEs) at 6 months in the IT (blue, *n* = 40) and IV (red, *n* = 21) arms of the trial. **b**, Proportion of patients experiencing at least one CTCAE treatment-related adverse event of grade 3 or 4 during the entire study in the IT (blue) and IV (red) arms of the trial. **c**, Treatment-related grade 3 or 4 toxicity event-free survival (EFS), defined as the time from inclusion randomization to first documentation of a treatment-related grade 3 or 4 toxicity. **d**, Distribution of the highest grade per patient of treatment-related toxicities according to treatment arm. **e**, Intra-individual cumulative toxicities with severity for each patient. Colour scheme as in **d**. **f**, Proportion of patients with more than one TRAE of grade 2 or higher. **g**, Peak and residual serum titrations of ipilimumab (left) and nivolumab (right) after the first, second and fourth cycle of treatment. Mann–Whitney unpaired analysis. Outlined numbers indicate mean values.

The primary objective of the study was to evaluate 6-month treatment tolerance, defined as CTCAE v4.0 treatment-related grade 3 or 4 adverse event-free survival for intratumoural ipilimumab combined with intravenous nivolumab. The IV arm served as an internal control to contextualize safety outcomes, although the study was not powered for efficacy comparisons. Among the 40 patients treated in the IT arm, 37 were evaluable for the primary end-point, from whom 9 (24.3%) experienced a treatment-related grade 3 or 4 adverse event within 6 months, below the pre-specified 30% Fleming threshold. Accordingly, 75.7% of patients in the IT arm remained free of grade 3 or higher toxicity compared with 42.9% in the IV arm (Fig. 1a).

Over the entire follow-up, the cumulative incidence of grade 3 or 4 treatment-related adverse events was 32.5% in the IT arm and 66.6% in the IV arm, reaching a plateau beyond 12 months (Fig. 1b,c). Treatment-related adverse events were generally less frequent and less severe in the IT arm, where only one grade 4 event was reported, whereas all patients in the IV arm experienced treatment-related adverse events, including grade 3 events in 52% of cases and grade 4 events in 14% of cases (Fig. 1d,e). Cutaneous, general and gastrointestinal toxicities were the most frequent, and increased liver enzyme concentrations were the most common biological abnormalities (Extended Data Table 2). Of note, no adverse events of grade 3 or higher related to the intratumoural injection procedure were reported. Overall, about 86% of patients in the IV arm developed multiple treatment-related adverse events of grade 2 or higher (Fig. 1f).

To assess systemic drug exposure, serum concentrations of ipilimumab and nivolumab were measured in 24 patients with serial samples available. Ipilimumab peak and trough levels were significantly lower in the IT arm (*P* < 0.0001), with mean peak concentrations of 2.2 ± 1.7 µg ml^−1^ versus 42.2 ± 12.3 µg ml^−1^ and mean trough concentrations of 0.9 ± 0.5 µg ml^−1^ versus 8.4 ± 10 µg ml^−1^ in the IT and IV arms, respectively. Unexpectedly, nivolumab plasma levels were significantly lower beyond cycle 2 in patients receiving intravenous ipilimumab compared with those treated with intratumoural ipilimumab, a difference that was not attributable to early nivolumab discontinuation (Fig. 1g).

## Intratumoural anti-CTLA4 increases local efficacy

**Fig. 2:**
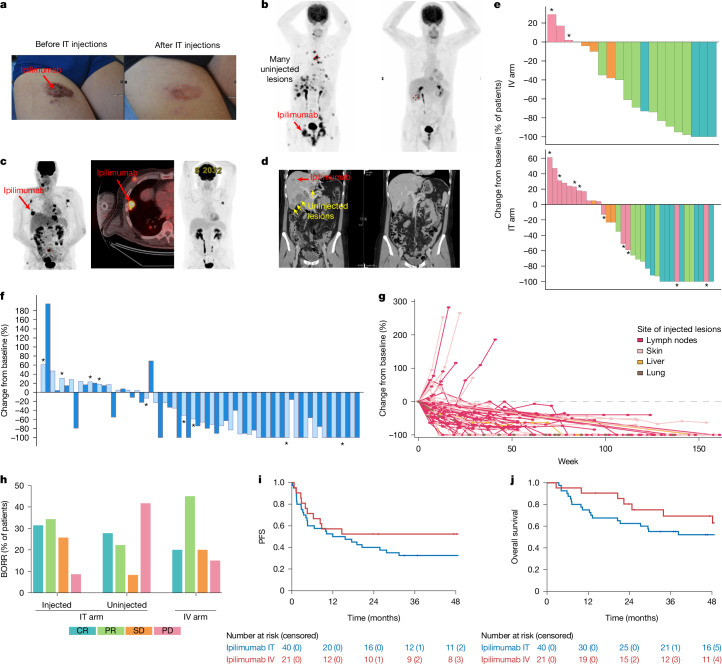
Efficacy of intratumoural ipilimumab in combination with intravenous nivolumab. **a**–**d**, Effects of treatment on patients in the IT arm; lesions injected with ipilimumab are indicated with a red arrow. **a**, Photograph showing the loco-regional response of a superficial skin lesion on the right thigh to intratumoural (IT) injections of ipilimumab. **b**, ^18^F-Fluorodeoxyglucose positron emission tomography (FDG-PET) images showing local and distant responses of multiple lesions to ipilimumab injections of a metastatic lymph node in the right groin. **c**, FDG-PET images of local and distant lesions before (left) and after (right) ipilimumab injection of a macroscopic metastatic lung lesion visible on PET (middle). **d**, An abdominal frontal computed tomography scan showing liver metastases before (left) and after (right) intratumoural ipilimumab injection of a single subcapsular hepatic tumour. **e**, Waterfall plots showing the BORR of target (uninjected) tumour lesions according to RECIST 1.1 criteria from patients in the IV and IT treatment arms. Asterisks indicate patients presenting with new lesions. **f**, Double waterfall plot illustrating the objective responses of injected (dark blue, left of each pair) versus uninjected (light blue, right of each pair) lesions according to RECIST 1.1 in a paired manner for individual patients, ordered from left to right according to depth of response of uninjected lesions. Asterisks indicate patients presenting with new lesions. **g**, Spider plot depicting the depth and kinetics of responses of injected lesions according to their location. **h**, Bar chart showing the BORR according to RECIST 1.1 between injected and uninjected (target) lesions in the IT and IV treatment arms. **i**,**j**, Progression-free survival (PFS) (**i**; log-rank test, *P* = 0.2315) and overall survival (**j**; log-rank test, *P* = 0.1808) estimations according to the Kaplan–Meier estimator. The median PFS for the IT arm was 13.8 months [4.4–27.7]. Median PFS was not reached for the IV arm. Median overall survival was not reached for the two treatment arms.

Notable antitumour activity of intratumoural ipilimumab was observed on injected lesions, with several patients achieving complete metabolic and radiological responses, including in visceral sites (Fig. 2a–d). Regression of injected lesions was frequently accompanied by responses in distant uninjected lesions, consistent with a systemic antitumour effect.

**Extended Data Fig. 2:**
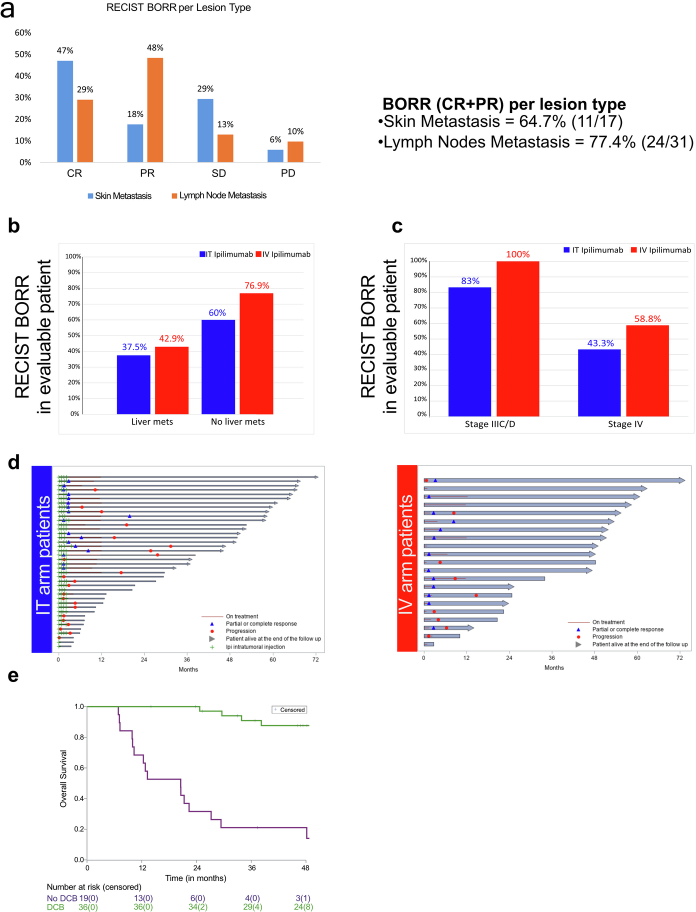
Clinical Response and Survival Outcomes Following Intratumoral versus Intravenous Ipilimumab Administration. **a**. Best Overall Response Rate (BORR) according to RECIST 1.1 per injected lesion types. **b**. BORR among patients with and without liver metastases (left), and **c**. among patients with Stage IIIC/D (left) or stage IV (right) in the IT (blue) and IV (red) arms. **d**. Swimmer plots for IT and IV arms showing patient follow-up, treatment duration and response over time. **e**. Kaplan Meier curves estimating the overall survival rate among patients with DCB and No DCB independently from the ipilimumab route of administration.

Best overall responses rates (BORRs) according to RECIST v1.1 criteria are summarized in waterfall plots for both treatment arms (Fig. 2e). In the IT arm, the BORR on evaluable injected lesions was 65.7%, including complete responses in 11 out of 35 patients (31.4%) and partial responses in 12 out of 35 patients (34.3%). On baseline uninjected RECIST target lesions from the same patients, complete and partial responses were observed in 25% (10 out of 40) and 20% (8 out of 40) of patients, respectively. Responses on injected lesions were generally deep and durable, and were often concordant with responses observed on uninjected lesions (Fig. 2f–g). Response rates differed according to lesion type, with higher response rates in lymph node than in skin metastases, whereas skin lesions showed higher complete response rates (Extended Data Fig. 2a). Among patients treated with deep-seated lesions, two achieved complete responses and one achieved stable disease.

In the IV arm, the BORR was 62%, including complete responses in 19% of patients and partial responses in 43% of patients (Fig. 2h). Baseline lactate dehydrogenase level and neutrophil-to-lymphocyte ratio were not associated with response. Although response rates appeared to be higher in the IV arm among patients without liver metastases, the trial was not powered for formal efficacy comparisons between treatment arms (Extended Data Fig. 2b,c).

After a median follow-up of 55.5 months (interquartile range (IQR): 48.2–62.8), median overall survival was estimated to be 50 months in the IT arm and was not reached in the IV arm, with no statistically significant differences in progression-free or overall survival between treatment arms (Fig. 2i,j). Durable clinical benefit (DCB), defined as maintenance of RECIST response or stable disease beyond 6 months, was associated with prolonged survival irrespective of the route of anti-CTLA4 administration (Extended Data Fig. 2d,e).

## Anti-CTLA4 and anti-PD1 pharmacodynamics

**Fig. 3:**
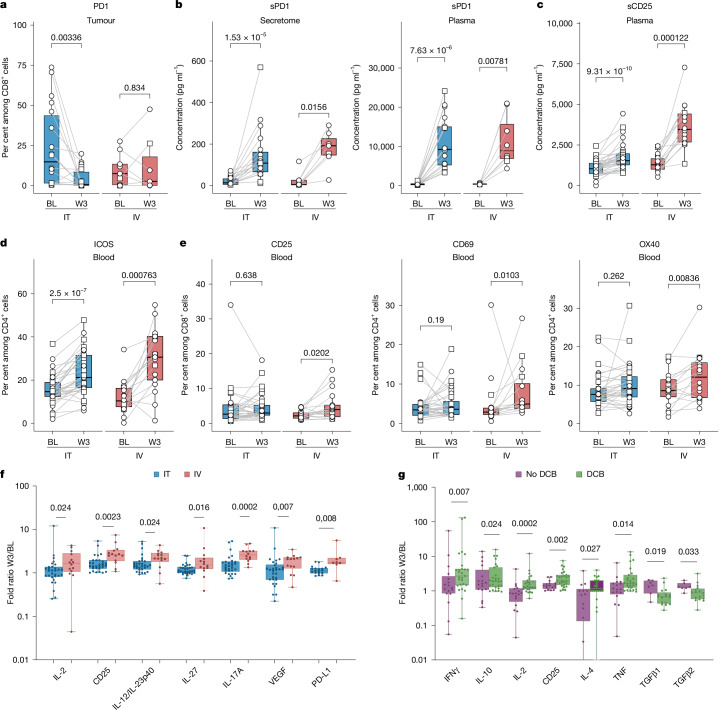
Pharmacodynamic effects of anti-CTLA4 plus anti-PD1 therapy. **a**, Fresh tumour biopsy analysis by flow cytometry of the proportions of PD1-positive cells among CD8^+^ T cells in the IT (*n* = 18) and IV (*n* = 7) arms between baseline (BL) and week 3 of treatment (W3). **b**, Soluble PD1 (sPD1) concentrations in the supernatant of the tumour biopsies (secretome) and in plasma. Secretome: IT arm, *n* = 17, IV arm, *n* = 7; plasma: IT arm, *n* = 18, IV arm *n* = 8. **c**, Plasma sCD25 concentrations. **d**, Proportions of ICOS^+^ cells among CD4^+^ T cells in fresh whole blood from patients treated in the IT (*n* = 32) and IV (*n* = 16) arms. **e**, Proportions of CD8^+^ T cells expressing CD25 (left), CD4^+^ T cells expressing CD69 (middle) and CD4^+^ T cells expressing OX40 (right) in fresh whole blood from patients in the IT and IV treatment arms. CD8^+^CD25^+^: IT arm, *n* = 32, IV arm, *n* = 17; CD4^+^CD69^+^ and CD4^+^OX40^+^: IT arm, *n* = 32, IV arm, *n* = 15. **f**, Significant fold changes in plasma concentration (week 3 versus baseline) of cytokines, chemokines and soluble factors in patients treated in the IT (*n* = 30, except PD-L1, *n* = 18) and IV (*n* = 14, except PD-L1, *n* = 8) arms. **g**, Significant fold change (week 3 versus baseline) in plasma concentration of cytokines, chemokines and soluble factors in patients with no DCB (*n* = 17 except IL-4, *n* = 14; TGFβ1 and TGFβ2, *n* = 7) or with DCB (*n* = 27 except IL-4, *n* = 22; TGFβ1 and TGFβ2 *n* = 17). **a**–**g**, Two-sided Wilcoxon test without multiple test adjustment. In box plots in all figures, the centre line represents the median, box bounds represent 25th and 75th percentiles, and whiskers extend to 1.5 times the interquartile range from the box bounds.

**Extended Data Fig. 3:**
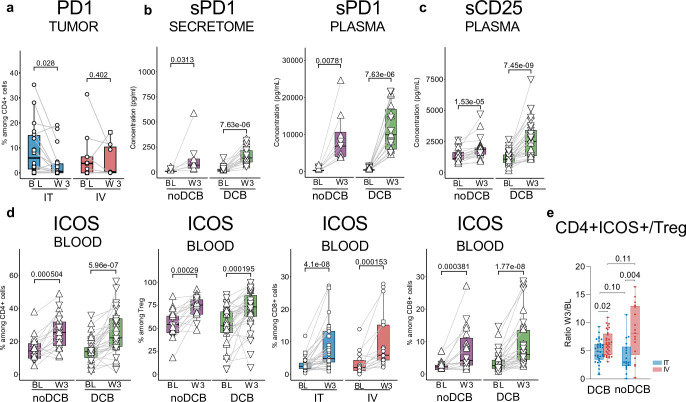
Pharmacodynamic markers of anti-CTLA4 and anti-PD1 according to clinical benefit. **a**. Proportion of PD-1^+^ cells among CD4^+^ T cells in fresh tumor biopsies assessed by flow cytometry in patients treated in the intratumoral (IT, n = 16) and intravenous (IV, n = 7) arms at baseline (BL) and week 3 (W3). **b**. Soluble PD-1 concentrations in plasma (left) and tumor biopsy supernatants (secretome; right) at BL and W3 in patients with subsequent durable clinical benefit (DCB) or no durable clinical benefit (noDCB). **c**. Plasma concentrations of soluble CD25 (sCD25) at BL and W3 in patients with subsequent DCB (n = 26) or noDCB (n = 17). **d**. Proportion of ICOS^+^ cells among CD4^+^ T cells in fresh whole blood according to clinical outcome (DCB, n = 31; noDCB, n = 17; left), in circulating Tregs (middle left), and among CD8^+^ T cells stratified by treatment arm (IT, n = 16; IV, n = 7; middle right) or by subsequent clinical outcome (right) at BL and W3. **e**. Ratio of circulating CD4^+^ICOS^+^ cells to Tregs in patients treated in the IT (blue) or IV (red) arms, stratified by subsequent DCB (IT, n = 18; IV, n = 11) or noDCB (IT, n = 14; IV, n = 3). **a**–**e** Two-sided Wilcoxon tests without multiple-testing correction.

Patients underwent serial blood sampling and tumour biopsies, including fresh tissue analyses, at baseline and on treatment to explore pharmacodynamic effects (sampling schedule in Extended Data Fig. 1b; sample manifest in Extended Data Table 3a,b). PD1 membrane expression in tumour biopsies was monitored using a non-competing anti-PD1 staining clone. PD1 expression decreased on CD4^+^ and CD8^+^ tumour-infiltrating lymphocytes in injected lesions following intratumoural anti-CTLA4 administration (Fig. 3a and Extended Data Fig. 3a).

Consistent with PD1 target engagement, soluble PD1 levels increased markedly in tumour secretome and plasma in the two treatment arms (Fig. 3b), independently of clinical outcome (Extended Data Fig. 3b). Similarly, systemic pharmacodynamic effects classically associated with anti-CTLA4 therapy were observed even with low-dose intratumoural ipilimumab. Soluble CD25 increased in both treatment arms (Fig. 3c), also independently of outcome (Extended Data Fig. 3c). ICOS expression was upregulated on circulating CD4^+^ T cells, including regulatory T cells, as well as on CD8^+^ T cells (Fig. 3d and Extended Data Fig. 3d), without association between the CD4^+^ICOS^+^ cell/T_reg_ cell ratio and clinical outcome (Extended Data Fig. 3e).

Additional pharmacodynamic changes were preferentially observed in patients receiving systemic high-dose ipilimumab, including increased activation markers on circulating T cells, such as CD25 on CD8^+^ T cells and CD69 and OX40 on CD4^+^ T cells (Fig. 3e).

Out of 35 cytokines, chemokines and soluble factors assayed in the blood, several increased between baseline and on treatment but the fold ratios of these increases were significantly higher in the IV arm only for IL-2, soluble CD25 (sCD25; CD25 is also known as IL-2Rα), IL-12/IL-23p40, IL-27, IL-17A, VEGF and soluble PD-L1 (Fig. 3f). In patients with subsequent durable clinical benefit (DCB), increases of these ratios were found specifically for IFNγ, IL-10, IL-2, sCD25, IL-4 and TNF and decreases of these ratios were found for TGFβ1 and TGFβ2 (Fig. 3g). Overall, strong pharmacodynamic evidence of PD1 and CTLA4 target engagements were found in both treatment arms of the trial with more pronounced systemic immune activation in patients receiving high-dose intravenous anti-CTLA4 therapy. However, circulating levels of certain cytokines increased only in patients with subsequent DCB, regardless of the route of anti-CTLA4 administration.

## Effects of somatic genomic alterations on outcomes

**Fig. 4:**
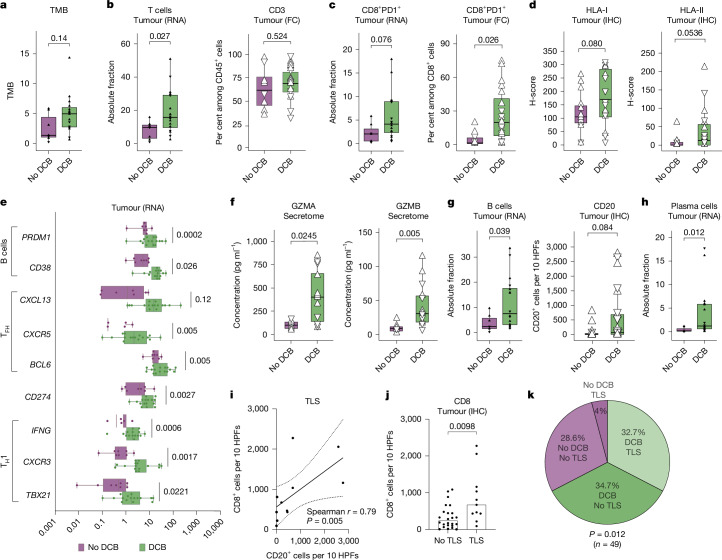
MHC-I and MHC-II-mediated tumour immunity are required for DCB. **a**, Tumour mutational burden (TMB) estimated by WES in baseline tumour biopsies from patients with DCB (*n* = 16; green) or patients with no DCB (*n* = 7; purple). **b**, Baseline absolute fraction of intratumoural T cells inferred by transcriptomic deconvolution (RNA-seq; left; no DCB, *n* = 7; DCB, *n* = 16) and proportion of CD3^+^ T cells among CD45^+^ cells identified by flow cytometry (FC) (right; no DCB, *n* = 8; DCB, *n* = 19) in fresh tumour biopsies. **c**, Absolute fraction of CD8^+^PD1^+^ cells identified by RNA-seq (left; no DCB, *n* = 7; DCB, *n* = 16) and proportion of CD8^+^PD1^+^ cells identified by flow cytometry (right; no DCB,* n* = 8; DCB, *n* = 19) in baseline tumour biopsies. **d**, HLA-I (left) and HLA-II (right) proteins detected by IHC in baseline tumour biopsies from patients with no DCB (*n* = 14) or patients with DCB (*n* = 18). **e**, Baseline tumour gene expression of markers from T_H_1 cells, T_FH_ cells and plasma B cells, and *CD274* expression (encoding PD-L1) by RNA-seq analysis of samples from patients with no DCB (*n* = 8) or patients with DCB (*n *= 16). **f**, GZMA and GZMB concentrations in the secretome of baseline tumour biopsies (no DCB, *n* = 6; DCB, *n* = 12). **g**, Absolute fraction of B cells identified by RNA-seq (left; no DCB, *n* = 7; DCB, *n* = 16) and density of CD20^+^ cells detected by IHC (right; range 0–2,769 cells per 10 high-power fields (HPFs)) in baseline tumour biopsies (no DCB, *n* = 14; DCB, *n* = 22). **h**, Absolute fraction of plasma B cells inferred by RNA-seq in baseline tumour biopsies (no DCB, *n* = 7; DCB, *n* = 16). **i**, Correlation of CD8^+^ and CD20^+^ cell densities (detected by IHC) in baseline tumour biopsies from patients with TLS (*n* = 11). The grey dotted line indicates the ordinary least-squares linear regression (mean estimate). **j**, CD8^+^ cell density in baseline tumour biopsies from patients with (*n* = 11) or without (*n* = 24) TLS. **k**, Proportion of tumours containing TLS at baseline in patients with no DCB or or patients with DCB. **a**–**h**, Two-sided Wilcoxon test without multiple test adjustment.

**Extended Data Fig. 4:**
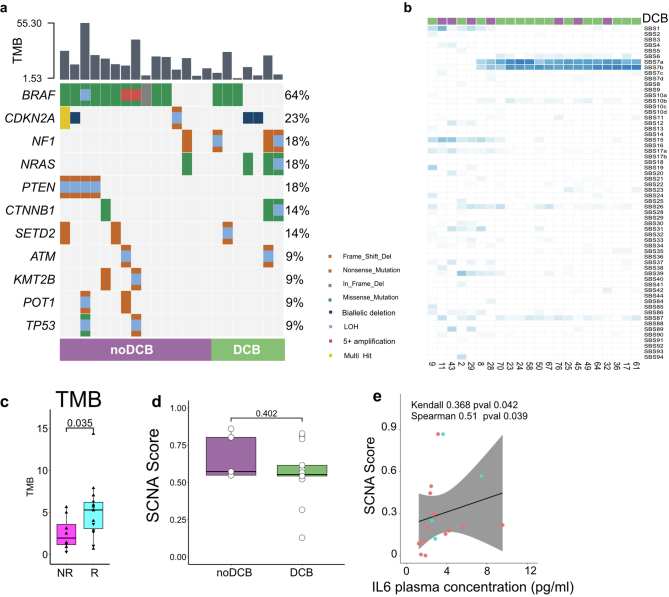
Whole Exome sequencing and Somatic Genomic Alterations according to the clinical benefit. **a**. Oncoplot derived from whole-exome sequencing (WES) of tumor biopsies, showing missense mutations (green), protein-disrupting mutations (brown), loss of heterozygosity (LOH; light blue), biallelic deletions (dark blue), double mutations (yellow), in-frame deletions (grey), and focal amplifications (red). **b**. Single-base substitution (SBS) mutational signatures derived from WES data. SBS7a and SBS7b signatures, characterized predominantly by C > T substitutions at TCN and CCN contexts, respectively, are indicative of ultraviolet (UV) light exposure. **c**. Tumor mutational burden (TMB) in patients classified as RECIST v1.1 responders (R) or non-responders (NR). **d**. Baseline comparison of somatic copy number alteration (SCNA) scores between patients with no durable clinical benefit (noDCB, n = 7) and durable clinical benefit (DCB, n = 13). **e**. Correlation between SCNA score and plasma IL-6 concentrations, with DCB patients shown in red and noDCB patients in blue; the grey shaded area denotes the 95% confidence interval of the fitted model. **c–e**. Two-sided Wilcoxon tests without multiple-testing correction.

Whole-exome sequencing (WES) confirmed that tumour samples from the cohort carried typical melanoma-associated genomic alterations (Extended Data Fig. 4a), with most tumours displaying the ultraviolet mutational signature (Extended Data Fig. 4b). Tumour mutational burden was higher in patients with objective responses (Extended Data Fig. 4c), but there was no substantial difference in mutational burden between patients with DCB and those with no DCB (Fig. 4a). Similarly, the number of somatic copy-number alterations (SCNAs) did not differ between DCB and no-DCB tumours (Extended Data Fig. 4d), and only a modest correlation was observed between the number of SCNAs and circulating IL-6 concentration (Extended Data Fig. 4e).

Because systemic immune activation was observed across treated patients but did not uniformly translate to DCB, we next investigated tumour biological features associated with treatment outcome.

## DCB requires MHC-mediated tumour immunity

**Extended Data Fig. 5:**
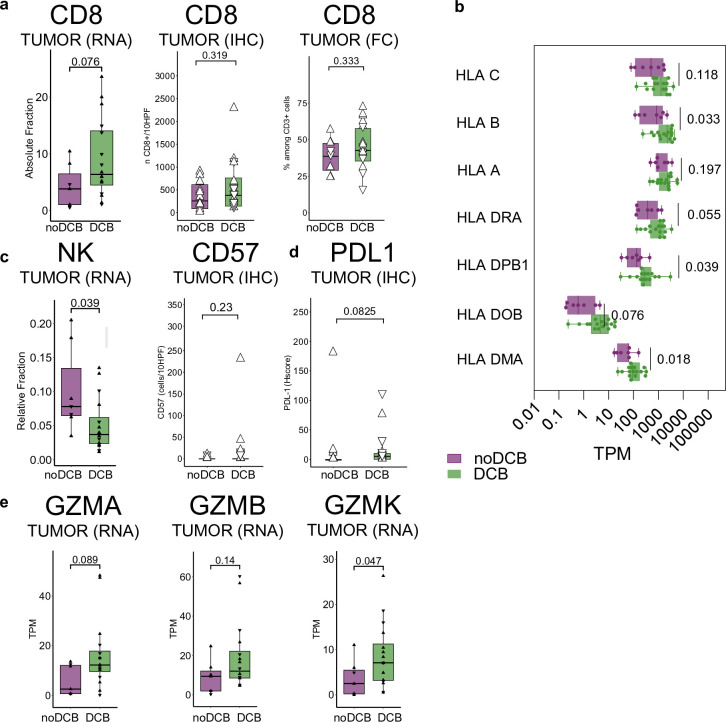
Immune Cytotoxic markers in patient with or without subsequent DCB. **a**. Absolute fraction of tumor CD8 T cells (left, RNAseq deconvolution) (DCB n = 16, noDCB n = 7), CD8 protein expression (middle, IHC) (DCB n = 20, noDCB n = 14), and tumor CD8 protein expression (right, flow cytometry) (DCB n = 16, noDCB n = 7), in tumors at baseline from patients with subsequent DCB or noDCB. **b**. Tumor gene expression of HLA-I and HLA-II molecules in baseline biopsies of patients with subsequent DCB (n = 16) or noDCB n = 7). **c**. Baseline tumor relative fraction of NK cells (DCB n = 16, noDCB n = 7) (left, RNAseq) and CD57 protein expression (DCB n = 22, noDCB n = 14) (right, IHC) in patients with subsequent DCB or noDCB. **d**. Baseline levels of PD-L1 protein expressions in tumors from patients with subsequent DCB (n = 20) or noDCB (n = 14), **e**. Tumor baseline gene expression (RNAseq) of granzymes A, B and K (GZMA, left; GZMB, middle; GZMK, right) from patients with subsequent DCB (n = 16) or noDCB (n = 7). **a**–**e**. Two-sided Wilcoxon test without multiple test adjustment.

Baseline tumour T cell infiltrates were higher in patients with subsequent DCB according to transcriptomic deconvolution, although not by flow cytometry (Fig. 4b). Total CD8^+^ T cell infiltration did not differ between groups across transcriptomic, immunohistochemistry (IHC) and flow cytometry analyses (Extended Data Fig. 5a). By contrast, both transcriptomic deconvolution and flow cytometry showed that CD8^+^PD1^+^ T cells were more abundant at baseline in tumours from patients with DCB (Fig. 4c).

To explore interactions between T cells and tumour cells, we assessed major histocompatibility complex class I (MHC-I) expression. A trend toward higher HLA-I protein expression was observed by IHC in baseline tumours from patients with DCB (Fig. 4d), with stronger differences in HLA-B alleles at the RNA level (Extended Data Fig. 5b). Lower HLA-I expression in no-DCB tumours was associated with a higher relative fraction of natural killer (NK) cells according to transcriptomic deconvolution, with no difference in absolute NK cell infiltration by IHC (Extended Data Fig. 5c).

**Fig. 5:**
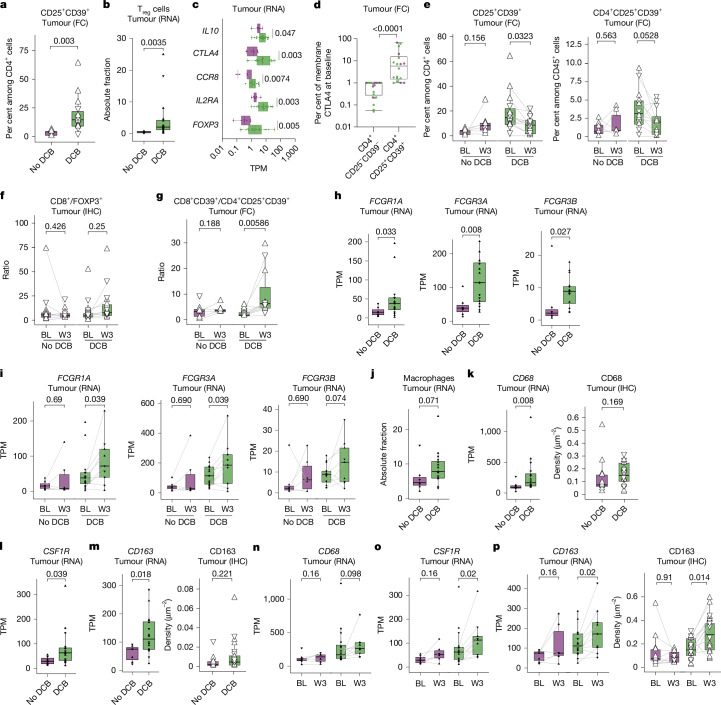
T_reg_ cells and M2 macrophages are abundant in tumours from patients with DCB. **a**, Proportion of CD39^+^CD25^+^ cells among CD4^+^ T cells (identified by flow cytometry) in baseline biopsies (DCB, *n* = 13; no DCB, *n* = 7). **b**, Absolute fraction of T_reg_ cells (inferred by RNA-seq) in baseline biopsies (DCB, *n* = 16; no DCB, *n* = 7). **c**, Baseline expression of T_reg_ cell-associated genes (from RNA-seq) in tumours (DCB, *n* = 18; no DCB, *n* = 7). *IL2RA* encodes CD25. TPM, transcripts per million. **d**, Membrane CTLA4 expression on CD4^+^CD25^−^CD39^−^ (*n* = 20) and CD4^+^CD25^+^CD39^+^ (*n* = 18) cells at baseline (detected by flow cytometry) in biopsies from patients with DCB (green) or no DCB (purple). **e**, Proportion of intratumoural CD25^+^CD39^+^ T_reg_ cells (left) and CD4^+^CD25^+^CD39^+^ cells (right) among total CD45^+^ cells (detected by flow cytometry) at baseline and at week 3 of treatment (DCB, *n* = 13; no DCB, *n* = 7). **f**, Ratio of CD8^+^ cells to FOXP3^+^ cells (detected by IHC; range 0–73.23) in tumours (DCB, *n* = 18; DCB, *n* = 14). **g**, Ratio of intratumoural CD8^+^CD39^+^ cells to CD4^+^CD25^+^CD39^+^ cells (detected by flow cytometry) (DCB, *n* = 12; no DCB, *n* = 6). **h**, Baseline FcγR gene expression in tumours (DCB, *n* = 16; no DCB, *n* = 7). **i**, *FCGR1A*, *FCGR3A* and *FCGR3B* expression in tumours (DCB, *n* = 16; no DCB, *n* = 7). **j**, Fraction of macrophages (inferred by RNA-seq) in baseline biopsies (DCB, *n* = 16; no DCB, *n* = 7). **k**, *CD68* gene expression (RNA-seq; DCB, *n* = 16; no DCB, *n* = 7) and density of CD68^+^ cells (IHC; DCB, *n* = 20; no DCB, *n* = 13) in baseline biopsies. **l**, *CSF1R* expression (from RNA-seq) in baseline biopsies (DCB, *n* = 16; no DCB, *n* = 7). **m**, *CD163* gene expression (RNA-seq; DCB, *n* = 16; no DCB, *n* = 7) and density of CD163^+^ cells (IHC; DCB, *n* = 20; no DCB, *n* = 13) in baseline biopsies. **n**–**p**, Gene expression of *CD68* (**n**), *CSF1R* (**o**) and *CD163* (**p**) and density of CD163^+^ cells (genes: DCB, *n* = 16; no DCB, *n* = 7; protein: DCB, *n* = 20; no DCB, *n* = 13) in tumours at baseline and at week 3 of treatment. **a**–**p**, For paired analyses, only patients with measurements at both time points were included in the Wilcoxon test, whereas all available patients are shown in graphs; centre values represent medians, and all comparisons were performed using two-sided Wilcoxon tests without multiple-testing correction.

Major histocompatibility complex class II (MHC-II)-mediated tumour immunity appeared to be more strongly associated with outcome. Pan-HLA-II protein expression and multiple MHC-II transcripts were higher at baseline in tumours from patients with DCB (Fig. 4d and Extended Data Fig. 5b). Consistently, expression of markers of T helper 1 (T_H_1) CD4^+^ T cell responses, including *TBX21* and *CXCR3*, as well as *IFNG*, were increased (Fig. 4e). This baseline IFNγ pathway activity was accompanied by increased expression of PD-L1 at gene and protein level (Fig. 4e and Extended Data Fig. 5d). Evidence of functional cytotoxic immunity was supported by higher levels of GZMA and GZMB in tumour secretomes and RNA-sequencing (RNA-seq) signatures, including GZMK (Fig. 5f and Extended Data Fig. 5e).

Follicular helper T (T_FH_) cells were also enriched in tumours from patients with DCB, as indicated by higher expression of *BCL6*, *CXCR5* and *CXCL13* (Fig. 4e). Consistent with this T_FH_ cell biology, B cell-mediated adaptive immunity was more prominent in DCB tumours, with higher B cell transcriptomic signatures, increased CD20 protein expression, and enhanced plasma-cell differentiation reflected by CD38 and PRDM1 expression (Fig. 4g–h). CD8^+^ T cell infiltration correlated with CD20^+^ B cells by IHC (Fig. 4i), and tumours containing tertiary lymphoid structures (TLSs) showed increased CD8^+^ T cell infiltration (Fig. 4j), although TLS detection lacked sensitivity despite high specificity for DCB status (Fig. 4k).

**Extended Data Fig. 6:**
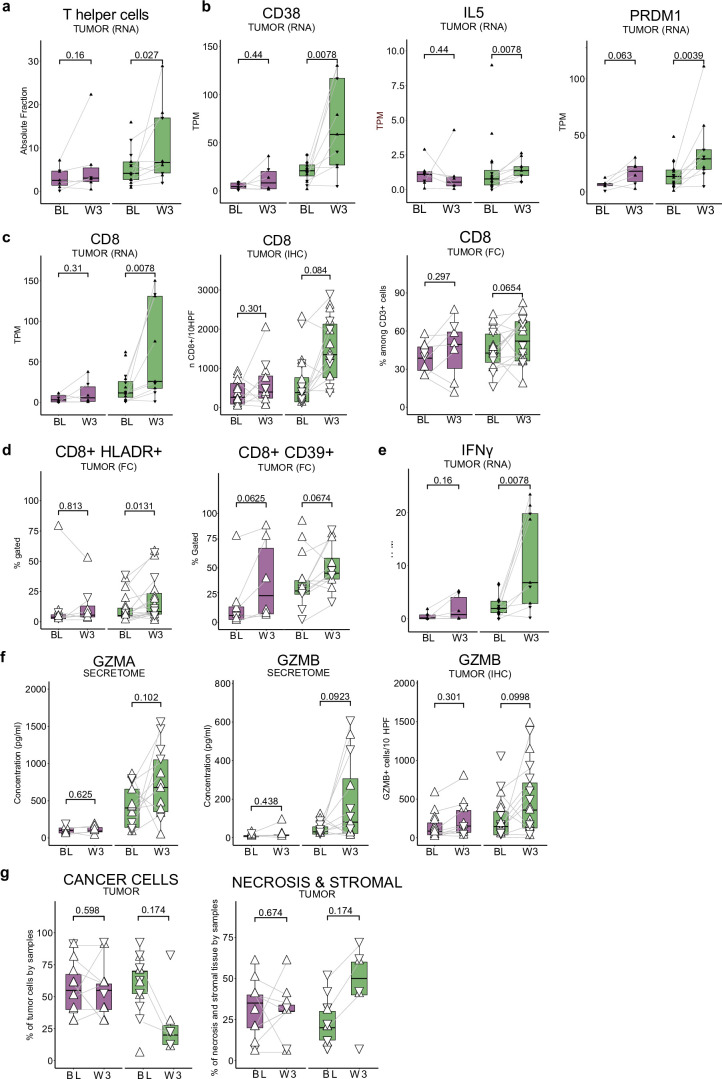
Immune dynamics upon treatment. **a**. T helper cells absolute fraction (RNAseq) between BL and W3 in patients with subsequent noDCB (n = 7) and DCB (n = 16). **b**. Expression (RNAseq) of the key plasma cells genes CD38 (left), IL5 (middle), PRDM1 (right) between BL and W3 in tumors from patients with subsequent DCB (n = 16) or noDCB (n = 7), **c**. Evolution of the CD8 gene expression (DCB n = 17, noDCB n = 7) (RNAseq; left), CD8 protein tissue expression (IHC; middle) (DCB n = 11, noDCB n = 9) and CD8 protein expression on CD3 + T-cells (DCB n = 17, noDCB n = 7) (flow cytometry; right) between BL and W3 in patients with subsequent DCB or noDCB, **d**. Evolution of the proportions of HLADR+ and CD39 + CD8 + T-cells between baseline (BL) and week 3 (W3) in patients with subsequent DCB (n = 11) or noDCB (n = 6), **e**. Gamma interferon gene expression (RNAseq) between baseline (BL) and week 3 (W3) in patients with subsequent DCB (n = 16) or noDCB (n = 7), **f**. Granzyme A protein concentration in the supernatant (secretome) of fresh tumor biopsies (DCB n = 11, noDCB n = 5) (left panel), Granzyme B protein concentration in the supernatant of fresh tumor biopsies (DCB n = 12, noDCB n = 5) (middle panel) and Granzyme B protein staining in fixed tumor biopsies (DCB n = 12; noDCB n = 9) (right panel), between BL and W3 in patients with subsequent DCB or noDCB, **g**. Proportions of cancer cells (left panel) and of necrotic and stromal cells (right panel) by IHC between BL and W3 in patients with subsequent DCB (n = 5) or noDCB (n = 9). **a**–**g**. Two-sided Wilcoxon test without multiple test adjustment.

These pre-existing immune features increased quantitatively three weeks after treatment initiation in patients with subsequent DCB but not in those without DCB. At week three, tumours from patients with DCB showed increased helper T cells, plasma cell markers and CD8^+^ T cell infiltration across transcriptomic, IHC and flow cytometry analyses (Extended Data Fig. 6a–c), including higher proportions of activated HLA-DR^+^ and CD39^+^CD8^+^ T cells (Extended Data Fig. 6d). This was accompanied by increased IFNγ expression and higher intratumoural granzyme levels (Extended Data Fig. 6e,f). Histologically, tumours from patients with DCB displayed reduced cancer cell content with increased necrosis and stromal tissue (Extended Data Fig. 6g).

Overall, patients who benefited from anti-PD1 plus anti-CTLA4 immunotherapy, irrespective of route of administration of ipilimumab, exhibited pre-existing MHC-I and MHC-II-dependent adaptive antitumour immunity that rapidly intensified after treatment initiation. We therefore examined immunosuppressive cell populations within the tumour microenvironment to understand resistance in patients without DCB.

## DCB is associated with intratumoural T_reg_ cells

Over the past two decades, tumour-infiltrating immunosuppressive cell subsets such as T_reg_ cells and M2 macrophages have been associated with poor outcomes in patients treated with conventional cytotoxic chemotherapies^19–22^. Their immunosuppressive functions contributed to the evolution of cancer immunology concepts, including immunosurveillance to immunoediting, highlighting the dynamic crosstalk between immune cell subsets that balance immune tolerance and tumour rejection during cancer development^23,24^.

**Extended Data Fig. 7:**
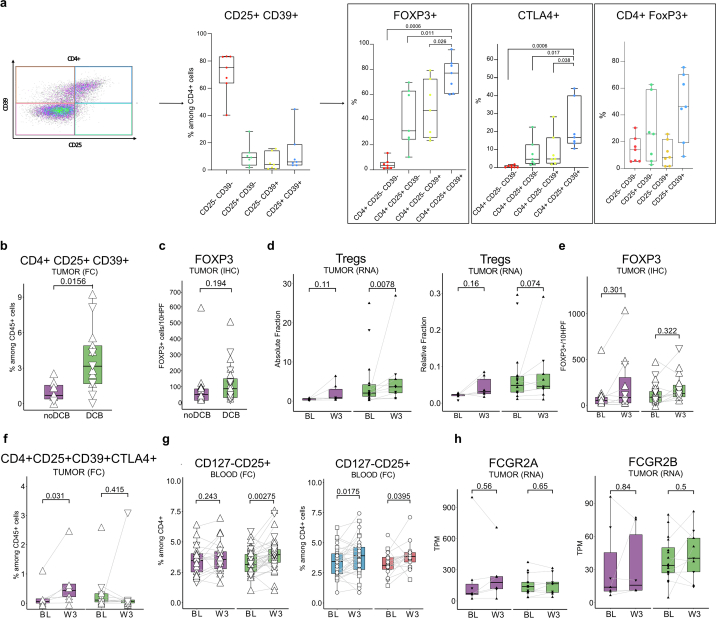
Intratumoral and Peripheral Tregs in patients upon treatment in IT and IV arm in patients with subsequent DCB or no DCB. **a**. Illustrative flow cytometry dot plot and relative proportions of CD25 +/- CD39 +/- and expression of intracellular FOXP3 and surface membrane CTLA4 proteins among CD4+ and CD4 + FOXP3 + T-cells (flow cytometry) within 7 melanoma fresh tumors from different donors, wo-sided Mann–Whitney U test without multiple test adjustment. **b**. Proportions of CD4 + CD25 + CD39+ among total CD45+ cells (flow cytometry) in baseline fresh tumor biopsies in patients with subsequent DCB (n = 13)or noDCB (n = 7), **c**. Total FOXP3 protein staining on baseline fixed tumor biopsies (IHC) in patients with subsequent DCB (n = 22) or noDCB (n = 14). **d**. Absolute (i.e taking into account total tumor cells; left panel) and relative (i.e normalized to the quantities of cancer cells; right panel) of regulatory T-cells (Tregs) gene signature by RNAseq deconvolution between BL and W3 in patients with subsequent DCB (n = 16)or noDCB (n = 7), **e**. Total FOXP3 protein staining on fixed tumor biopsies (IHC) between baseline (BL) and week 3 (W3) in patients with subsequent DCB (n = 11)or noDCB (n = 9), **f**. Proportions of CD4 + CD25 + CD39 + CTLA4+ cells among CD45+ at BL and on W3 in fresh tumor biopsies (flow cytometry) from patients with subsequent DCB (n = 11) or oDCB (n = 6), **g**. Circulating CD127-CD25+ among CD4 + T-cells (Tregs) in fresh whole blood (flow cytometry) between baseline and W3 from patients with subsequent DCB (n = 32) or noDCB (n = 17) (left panel) and in patients treated in the IT (n = 32) or IV (n = 17) arm (right panel), **h**. Gene expression of FCGR2A and FCGR2B (RNAseq) in frozen tumor biopsies between baseline and W3 from patients with subsequent DCB (n = 16) and noDCB (n = 7). **b**–**h**. Two-sided Wilcoxon test without multiple test adjustment.

Our team recently reported that clonally expanding tumour-infiltrating T_reg_ cells selectively co-express CD25 and CD39 and preferentially express membrane CTLA4 across cancer types, including melanoma^25^ (Extended Data Fig. 7a). Because 18-gauge fresh tumour biopsies yield insufficient cells for intracytoplasmic FOXP3 staining, activated intratumoural T_reg_ cells were tracked by CD25/CD39 double staining on tumour-infiltrating CD4^+^ T cells from prospectively collected fresh tumour biopsies analysed by flow cytometry (Extended Data Fig. 10).

Contrary to expectations, activated intratumoural T_reg_ cells were more abundant at baseline in patients with subsequent DCB. Increased proportions of CD25^+^CD39^+^CD4^+^ T cells were observed both within CD4^+^ T cells (Fig. 5a) and among CD45^+^ cells (Extended Data Fig. 7b). These findings were consistent with transcriptomic analyses showing increased T_reg_ cell deconvolution signatures (Fig. 5b), RNA-seq showing increased *FOXP3* gene expression and IHC showing increased FOXP3 protein expression (Fig. 5c and Extended Data Fig. 7c). Tumours from patients with DCB also displayed higher baseline expression of T_reg_ cell-associated markers, including CD25, CCR8, IL-10 and CTLA4 transcripts (Fig. 5c). In line with our previous work, membrane CTLA4 expression was enriched on CD25^+^CD39^+^CD4^+^ T cells, and there were no differences in membrane CTLA4 expression between patients with DCB and those with no DCB (Fig. 5d).

## Selective depletion of activated T_reg_ cells in DCB

Because these activated T_reg_ cells appeared to be the predominant cells expressing membrane CTLA4 proteins, we probed the changes that occurred to those cells upon treatment. First, we found that the absolute fraction of total T_reg_ cells (among total cells) per transcriptomic deconvolution showed a greater increase upon treatment than the relative fraction (excluding cancer cells) (Extended Data Fig. 7d). A similar trend, although not statistically significant, was found for total FOXP3 protein staining by IHC (Extended Data Fig. 7e). However, when we examined the subset of intratumoural CD4^+^CD25^+^CD39^+^ activated T_reg_ cells, we found a significant decrease in their proportions either within the CD4^+^ subset or within the total CD45^+^ subset (Fig. 5e). Of note, membrane protein expression of CTLA4 increased upon treatment in CD4^+^CD25^+^CD39^+^ T cells in patients with no DCB (Extended Data Fig. 7f). Overall, there was a trend for an increased intratumoural ratio of CD8^+^ cells over FOXP3^+^ cells in tumours of patients with DCB (Fig. 5f). Notably, the ratio of CD8^+^CD39^+^ tumour-specific T cells over CD4^+^CD25^+^CD39^+^ tumour-specific T_reg_ cells increased a median of threefold upon treatment in patients with DCB but remained unchanged in patients with no DCB (Fig. 5g). Of note, the blood levels of T_reg_ cells usually defined by the proportion of circulating CD25^+^CD127^−^CD4^+^ T cells did not decrease upon treatment, but rather increased, in patients with DCB with intratumoral or intravenous injection of ipilimumab (Extended Data Fig. 7g).

## Increased baseline FcγR and M2 macrophages in DCB

To better understand the differential effects of anti-CTLA4 therapy on CTLA4^+^ T_reg_ cells between patients with DCB and those with no DCB, and considering the potential role of FcγR-mediated T_reg_ cell depletion by Fc-competent IgG1 anti-CTLA4, we evaluated intratumoural FcγR expression. Baseline expression of *FCGR1A* (also known as *CD64*), *FCGR3A* (also known as *CD16A*) and *FCGR3B* (also known as *CD16B*) was higher in tumours from patients with DCB and increased further upon treatment (Fig. 5h,i), whereas expression of FCGR2A (also known as *CD32A*) and FCGR2B (also known as CD32b) did not differ between groups (Extended Data Fig. 7h).

Transcriptomic deconvolution revealed higher macrophage abundance at baseline in tumours from patients with DCB (Fig. 5j), which was confirmed by higher CD68 gene and protein expression (Fig. 5k). Markers of M2-polarized macrophages, including *CSF1R* and *CD163*, were also higher at baseline in DCB tumours (Fig. 5l,m). Upon treatment, expression of *CD68*, *CSF1R* and *CD163* expression further increased in tumours from patients with subsequent DCB but remained low in tumours from patients without DCB (Fig. 5n–p).

**Extended Data Fig. 8:**
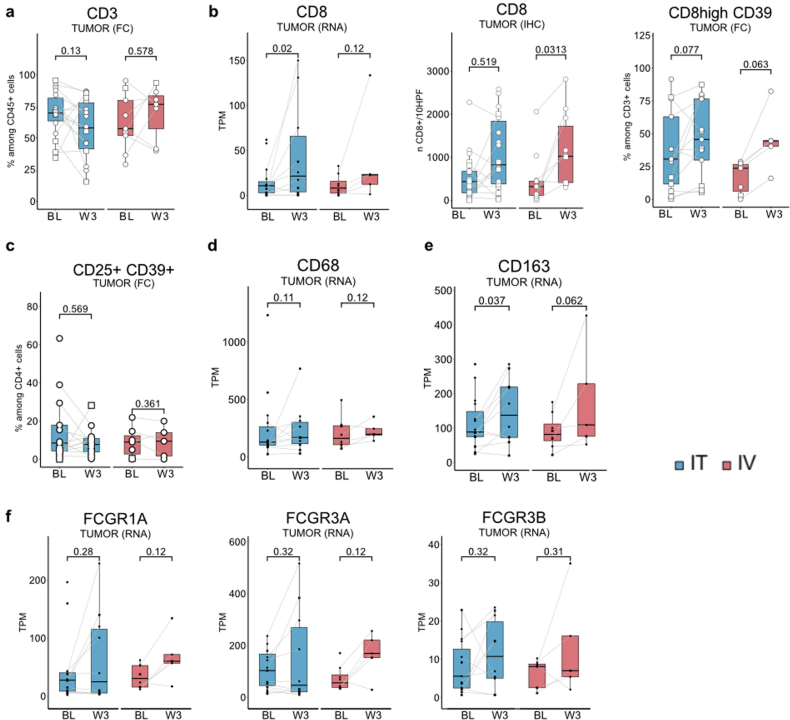
Tcells, Treg and Macrophages upon treatment in IT and IV arms. **a**. Fresh tumor biopsies analysis by flow cytometry of proportions of CD3 + T-cells from patients treated in the IT (n = 16) and IV (n = 7) arms between BL and W3. **b**. CD8 gene expression (RNAseq, left) between BL and W3 in patients treated in the IT (n = 15) and IV (n = 8) arms. CD8 protein expression (IHC, right, non paired analysis) between BL: IT n = 21; IV n = 13 and W3: IT n = 18, IV n = 10 in patients treated in the IT and IV arms. **c**. Proportions of CD8high CD39+ cells among total CD3+ cells at BL and W3 in the IT (n = 12) and IV (n = 5) arms. **d**. Proportions of CD4 + CD25 + CD39+ cells among total CD4+ at BL and on treatment (W3) in fresh tumor biopsies (flow cytometry) from patients treated in the IT (n = 12)and IV (n = 5) arms. **e**. CD68 gene expression (RNAseq) between BL and W3 from patients treated in the IT (n = 8) and IV (n = 7) arms. **f**. CD163 gene expression (RNAseq) between BL and W3 from patients treated in the IT (n = 8) and IV (n = 7) arms. **h**. FcGR1a (left), FcGR3a (middle) and FCGR3b (right) genes expression (RNAseq) between BL and W3 in patients treated in the IT (n = 8) and IV (n = 7) arms **a**–**f**. Two-sided Wilcoxon test without multiple test adjustment.

Despite the stronger systemic immune activation observed with high-dose intravenous ipilimumab, intratumoural immune cell composition did not differ according to the route of anti-CTLA4 administration. Total intratumoural T cell infiltration, T_reg_ cells and CD68 expression were similar between IT and IV arms (Extended Data Fig. 8a–d). Activated CD8^+^ T cells increased modestly upon treatment, most clearly detected by IHC and flow cytometry (Extended Data Fig. 8b). CD163 expression increased upon treatment in both treatment arms, together with a trend towards increased FcγR (Extended Data Fig. 8e,f).

## Discussion

Here, we demonstrate that intratumoural anti-CTLA4 at 0.3 mg kg^−1^ combined with intravenous anti-PD1 is safe in advanced melanoma, with a markedly reduced incidence of severe treatment-related adverse events compared with both the control IV arm and historical intravenous regimens^1^. The primary end-point was met, with a 22.6% cumulative incidence of grade 3 or higher treatment-related adverse events at 6 months, well below the predefined Fleming threshold. These results compare favourably with the 60% rate reported with standard-dose intravenous ipilimumab plus nivolumab^1^ and with the 33.9% rate observed in reduced-dose intravenous regimens^26^. In the context of emerging combination strategies incorporating anti-LAG3^27^, our findings support intratumoural anti-CTLA4 as a rational approach to maximize dose-dependent intralesional efficacy while mitigating systemic toxicity.

No local toxicity at injection sites, no grade 3 or higher procedure-related adverse events or grade 3 or higher gastrointestinal toxicity were observed in the IT arm, supporting the safety and feasibility of this approach. Accordingly, the ulcerations previously reported with intralesional ipilimumab combined with IL-2 were most probably attributable to IL-2, consistent with observations from intratumoural IL-2 monotherapy^28^.

Although most injected lesions were superficial, deeper lesions were not excluded by the protocol. Initial procedural caution limited their early use; however, as confidence in short-term and long-term safety increased, intratumoural ipilimumab was successfully administered to liver and lung metastases without additional safety signals and with preserved local efficacy. As reported with other intratumoural strategies, including T-VEC plus pembrolizumab (NCT02263508), injection procedures did not compromise patient accrual, which remained comparable to intravenous-only trials^29^.

Pharmacokinetic analyses showed that a fraction of intratumourally administered anti-CTLA4 reached the systemic circulation; however, peak serum ipilimumab concentrations were approximately 20-fold lower in the IT arm despite a tenfold lower administered dose, suggesting partial retention within injected tumours and/or their draining lymph nodes.

Although not powered for formal efficacy comparisons, objective response rates on RECIST target (uninjected) lesions and overall survival were lower in the IT arm (overall response rate 50%) than in the IV arm (overall response rate 65%). Notably, the control IV arm outperformed historical benchmarks for standard-dose intravenous ipilimumab plus nivolumab, in which the overall response rate was between 50.6% (Checkmate 511 trial) and 58% (phase 3 registration trial)^1^, probably reflecting the limited size of the IV arm cohort and residual imbalances despite stratified randomization. Conversely, the IT arm compared favourably with anti-PD1 monotherapy^7,30^ and with reduced-dose intravenous combination regimens, thereby supporting a potential systemic effect of intratumoral ipilimumab in addition to intravenous nivolumab^26^.

In the IT arm, high complete response rates were observed both in injected lesions (31%) and in uninjected target lesions (25%), exceeding those typically reported in melanoma trials^7,26^ and those observed in the IV arm. This finding supports a systemic component of intratumoural anti-CTLA4 activity. Two non-mutually exclusive mechanisms may account for these responses in uninjected (anenestic) lesions^31^. Local priming of antitumour immunity by intratumoural anti-CTLA4 can induce systemic immune responses, including at distant, uninjected sites, as demonstrated in preclinical models and supported by early clinical observations^14,32^. In parallel, a fraction of intratumourally administered anti-CTLA4 enters the systemic circulation and may directly contribute to immune modulation at distant tumour sites, consistent with experimental evidence showing comparable intratumoural accumulation after local or intravenous administration over time^33^. Finally, tumour-draining lymph nodes may have a critical role, as local delivery of low-dose anti-CTLA4 has been shown to induce immune remodelling of regulatory T cells and myeloid subsets both locally and systemically^34^.

Injection of ipilimumab (anti-CTLA4) into lesions achieved a very high objective response rate (65.7%), exceeding the response rate observed in uninjected lesions and formally demonstrating the added value of anti-CTLA4 beyond anti-PD1 therapy, with patients serving as their own internal controls for dose-dependent efficacy. These findings support the use of intratumoural ipilimumab (anti-CTLA4) in settings where local disease control is critical (such as oligometastatic and neoadjuvant contexts).

Systemic pharmacodynamic effects of ipilimumab were preserved in the IT arm despite the local administration at low doses. Intratumoural anti-CTLA4 induced robust upregulation of ICOS on circulating CD4^+^ and CD8^+^ T cells^35^, including T_reg_ cells, together with increased circulating soluble CD25^36^. These findings indicate that systemic CTLA4 pathway engagement can be achieved through local delivery, potentially via high local exposure within the tumour microenvironment and draining lymph nodes and/or low-level systemic diffusion, as pharmacodynamic readouts were comparable between intratumoural low-dose and intravenous high-dose administration. Some specific pharmacodynamic consequences of high-dose IV anti-CTLA4 were found to be the upregulation of immune activation markers on circulating T cells, especially CD69 on circulating CD4^+^ T cells. This heightened peripheral activation may account for the higher incidence of treatment-related adverse events observed with systemic dosing, and may potentially contribute to eradicating distant micrometastatic disease. We showed that the antitumour activity of combined anti-PD1 and anti-CTLA4 therapy is determined primarily by pre-existing tumour biology rather than by the route of anti-CTLA4 administration. Favourable outcomes were associated with higher baseline expression of MHC-I and MHC-II and with an abundance of adaptive immune effector cells. In particular, an ongoing cytotoxic immune response involving MHC-I-restricted CD8^+^ T cells, including PD1^+^ subsets, together with MHC-II-dependent T_H_1 CD4^+^ T cells expressing TBX21 and CXCR3, appeared to be required for clinical benefit, confirming the roles of CD8^+^ and CD4^+^ cytotoxic responses^37,38^.

We further identify a coordinated B cell response as an integral component of this antitumour immunity^39,40^, with higher baseline expression of T_FH_ and B cell markers in patients achieving DCB and a strong correlation between tumour-infiltrating CD8^+^ T cells and CD20^+^ B cells. This favourable immune context was amplified upon treatment in patients with DCB, notably through expansion of plasma B cells. Conversely, there was a substantial reduction of immune reactivity in patients with no DCB; notably there was a marked contrast in interferon responses between tumours from the DCB versus no DCB groups.

Unexpectedly, tumours from patients who subsequently achieved DCB had higher baseline levels of intratumoural T_reg_ cells and M2 macrophages. The coexistence of abundant cytotoxic effector cells with immunosuppressive populations supports the requirement for ongoing intratumoural immunoediting for responsiveness to combined anti-PD1 plus anti-CTLA4^41^. This observation supports the hypothesis that immune checkpoint-targeted therapies interfere in the crosstalk between cancer cells and the host immune system, reversing the excess of immune tolerance and fostering cancer immune rejection. This highlights that immune cell populations historically associated with poor prognosis under cytotoxic therapies, such as T_reg_ cells and M2 macrophages, may instead represent predictive markers for benefit from checkpoint blockade.

This observation also raises the controversial question of the mechanism of action of anti-CTLA4 in human tumours, particularly with respect to T_reg_ cell targeting. In mouse models, the antitumour efficacy of anti-CTLA4 requires Fc-competent, depleting isotypes and is associated with the elimination of activated, tumour-specific, CTLA4-expressing intratumoural T_reg_ cells through antibody-dependent cellular cytotoxicity and phagocytosis mediated by FcγR-positive myeloid cells^42^. In humans, evidence for intratumoural T_reg_ cell depletion has remained controversial, probably owing to methodological limitations and differences in sample timing.

In Humans, although two clinical trials supported T_reg_ cell depletion upon anti-CTLA4 monotherapy^12,43^, there has been controversy about the ability of monoclonal anti-CTLA4 to deplete intratumoural T_reg_ cells, supported by a retrospective analysis^44^.

Our data support a unifying model in which these discrepancies arise from the need to specifically assess activated intratumoural T_reg_ cells. High membrane CTLA4 expression, a prerequisite for Fc-mediated depletion, is largely restricted to activated T_reg_ cells and can only be reliably detected by flow cytometry, and thus only on fresh tumour samples. Second, only a subset of activated T_reg_ cells upregulate CTLA4 at their membrane upon TCR engagement, and these cells can be identified by flow cytometry with CD25 and CD39 double staining of tumour-infiltrating CD45^+^CD3^+^CD4^+^ T cells, in which expression of CD39, an ectoenzyme of the immunosuppressive adenosine pathway, is a marker of TCR engagement^45–49^.

Using this approach, we demonstrate that the number of CD4^+^CD25^+^CD39^+^ activated T_reg_ cells was selectively decreased upon treatment in patients with DCB, concomitant with high intratumoural expression of activating FcγR (CD16A, CD16B and CD64). By contrast, non-responding patients lacked FcγR-rich effector cells, did not deplete activated T_reg_ cells, and instead exhibited increased CTLA4 membrane expression on this subset upon treatment. Together, these findings support the conclusion that, as in mice, anti-CTLA4 antibodies can deplete activated tumour-specific T_reg_ cells in humans, but only in tumours that contain both activated T_reg_ cells and FcγR-positive effector cells.

Another important finding of our study is the potential of analyses of fresh tumour samples to identify predictive biomarkers of response to immunotherapy. In contrast to current biomarkers that rely on fixed or frozen tumour materials, fresh tumour analyses enable rapid, highly sensitive and biologically informative assessments within hours of sampling. Techniques such as flow cytometry and multiplex soluble factor quantification enable direct evaluation of immune checkpoint expression on defined cellular subsets, target saturation and engagement, and dynamic immune activation. In this context, quantification of CD4^+^CD25^+^CD39^+^ T cells by flow cytometry and measurement of intratumoural granzymes in the tumour secretome represent simple and rapid approaches to forecast therapeutic benefit. As these technologies are routinely used for haematologic malignancies, they could be readily translated to solid tumours, although prospective validation in independent cohorts will be required.

**Extended Data Fig. 9:**
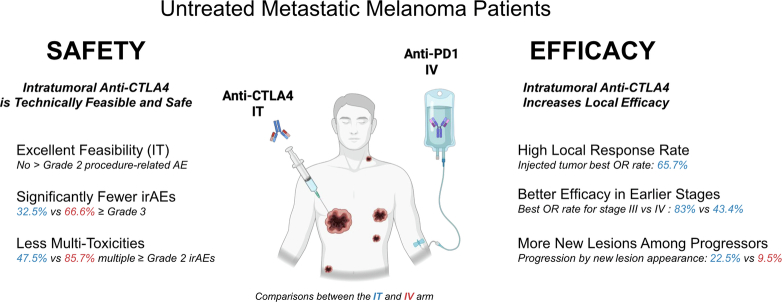
Safety and Efficacy Trade-offs of Intratumoral Anti-CTLA and Intravenous Anti-PD1 as First-Line Treatment for Advanced Melanoma Patients. Graphical summary depicting the safety and efficacy profile of intratumoral anti-CTLA4 + IV anti-PD1.

In summary, this trial met its primary end-point, with a lower incidence of grade 3 or 4 treatment-related adverse events for intratumoural anti-CTLA4 combined with intravenous anti-PD1 compared with standard intravenous dual checkpoint blockade, achieving efficacy comparable to anti-PD1 monotherapy. We establish the feasibility and safety of intratumoural anti-CTLA4 in metastatic melanoma, including for deep-seated lesions, and demonstrate enhanced efficacy on injected tumours, with potential activity on distant uninjected lesions relative to anti-PD1 monotherapy controls (Extended Data Fig. 9).

Analysis of fresh tumour biopsies enabled the identification of rapid, biologically grounded biomarkers predictive of response to combined checkpoint blockade. Therapeutic benefit was associated with the coexistence of activated T_reg_ cells and M2 macrophages alongside MHC-I and MHC-II restricted CD8^+^, CD4^+^ and B cell-mediated adaptive immunity, supporting the idea that ongoing intratumoural immunoediting is a prerequisite for effective combined anti-PD1 plus anti-CTLA4 therapy. Together, these findings highlight the clinical potential of intratumoural immunotherapy in oligometastatic and neoadjuvant settings, not only in melanoma but also in other checkpoint-sensitive tumours, offering opportunities to enhance efficacy while reducing toxicity. They further provide a strong rationale for biologically driven patient selection and stratification in future prospective trials aimed at personalizing immunotherapy and addressing primary resistance.

## Methods

### Study sponsorship, authorizations and ethics

This study was an investigator initiated trial sponsored by Gustave Roussy and covered by a Biomedical Research Promoter Civil Liability insurance contract (contract 124.895) in accordance with the provisions of the French law (Decree 2006-477 of 26 April 2006 and Article L.1121-10 of the French Public Health Code). This study was approved by the French Health Agency (Agence Nationale de Sécurité du Médicament) on 29 March 2016 (ANSM 160104A-12) and by the national ethics committee (Comité de Protection des Personnes Ile-de-France VIII) on 16 February 2016 (CPP 160215). The study was registered on EUDRACT (2015-005429-37) on 30 November 2015 and on ClinicalTrials.gov (NCT02857569) on 19 July 2016. The study was conducted in accordance with the Declaration of Helsinki and international Conference on Harmonization Good Clinical Practice (GCP). All investigators were GCP certified. The study was approved by the Gustave Roussy institutional review board (CSET 2015/2334) on 24 November 2015. All patients provided written informed consent prior enrollment in the trial and before any study specific procedure for clinical data and ancillary analysis anonymous use. Ipilimumab and nivolumab were supplied by Bristol Myers Squibb.

### Objectives

The study aimed to evaluate the 6-month treatment tolerance defined as the treatment-related grade 3–4 adverse event-free survival of the combination therapy intratumoural (IT) ipilimumab plus intravenous (IV) nivolumab. The IV ipilimumab plus IV nivolumab (same doses as in phase 1) arm will be used as an internal control to interpret the results obtained in the IT ipilimumab arm.

Secondary objectives were to explore the types of toxicities generated by the combination therapy in the two treatment arms and to evaluate the efficacy of IT ipilimumab in combination with IV nivolumab.

Translational objectives were to demonstrate the lower systemic exposure to ipilimumab in the IT arm and to identify predictive biomarkers of response.

To assess response to treatment using several end-points for efficacy based either on RECIST 1.1, or on immune-related response criteria.

### Key eligibility criteria

Main inclusion and non-inclusion criteria are presented below (not exhaustive list, if needed cf. protocol).

#### Inclusion criteria

- Men and women ≥18 years of age who signed a written informed consent before any study-related procedure.
- Histologically confirmed and clinically or radiologically progressing unresectable stage III or stage IV melanoma, as per American Joint Committee on Cancer (AJCC) staging system.
- Patients with at least two lesions: at least one injectable tumour lesion (≥1 cm^3^) and at least one target lesion (measurable lesion as per RECIST 1.1). Disease measurable by computed tomography or MRI (RECIST 1.1).
- Good general status, Eastern Cooperative Oncology Group (ECOG) performance status of 0 or 1.
- First line treatment.
- Recent (less than 3 month) tumour tissue provided for patient stratification and biomarker analyses.
- Subjects with wild-type *BRAF*. Those with *BRAF* mutations can be included only if they have been treated with, or developed toxicity with or refused to be treated with, BRAF-and/or MEK-targeted therapy.
- Usual laboratory test limitations (refer to protocol).
- Usual limitations concerning women of childbearing potential and sexually active men.

#### Non-inclusion criteria

- Active brain metastases or leptomeningeal metastases.
- Ocular melanoma.
- Any serious or uncontrolled medical disorder.
- Prior malignancy active within the previous 3 years except for locally curable cancers.
- Subjects with active, known or suspected autoimmune disease.
- Subjects with a condition requiring systemic treatment with either corticosteroids (>10 mg daily prednisone equivalents) or other immunosuppressive medications within 14 days of study drug administration.
- Prior treatment with an anti-PD1, anti-PD-L1, anti-PD-L2, anti-CTLA4, or any other antibody or drug specifically targeting T cell co-stimulation or immune checkpoint pathways.
- Positive test for hepatitis B virus surface antigen (HBV sAg) or hepatitis C virus ribonucleic acid (HCV antibody) indicating acute or chronic infection, or HIV infection.
- Patients presenting coagulation abnormalities and/or patients requiring concomitant treatment with therapeutic doses of anticoagulants. Prophylactic low dose of anticoagulants for thrombo-embolic events is allowed. Prophylactic anticoagulants shall be stopped during 24 h before and after deep lesion biopsies/injections. No stopping required for biopsies/injections of skin and sub-cutaneous lesions.

### Treatment and follow-up scheduled

#### Experimental IT arm

A. Ipilimumab: 0.3 mg kg^−1^ IT injection every 3 weeks until complete response, eradication of all injectable sites, disease progression or toxicity, for a maximum of 4 doses (control: IV standard of care and marketing authorization). Pure ipilimumab from commercial vials at 5 mg ml^−1^ was used for IT injections. For instance, a patient weighing 75 kg would receive 75 × 0.3 = 22.5 mg—that is, 4.5 ml of pure ipilimumab into the chosen tumour lesions. All injections were performed under image guidance by interventional radiologists using 22 gauge (22G) Chiba needles with a single endhole. The injected volume ranged between 3 and 6.5 ml (at 5 mg ml^−1^). For large lesions, a different part of the tumour was injected at each cycle in a clockwise manner. A very low injection rate was used (1 ml min^−1^) to avoid reflux and optimize distribution within the tumour. Local anaesthesia with lidocaine (2%) was used for all except some superficial skin lesions. Minimal size of injected lesions was 1 cm in diameter per protocol. The volume of ipilimumab per patient could be divided in order to inject several lesions but with the objective of adjusting the injected volume to the volume of the treated lesion as much as possible.
B. Nivolumab: 1 mg kg^−1^, IV injection every 3 weeks during IT ipilimumab treatment period and 3 mg kg^−1^, IV injection every 2 weeks after IT ipilimumab treatment interruption. Treatment should be continued as long as clinical benefit is observed or until treatment is no longer tolerated by the patient, for a maximum of 12 months.

#### Standard arm

A. Ipilimumab: 3 mg kg^−1^, IV injection every 3 weeks for a maximum of 4 doses as per standard of care and marketing authorization.
B. Nivolumab: 1 mg kg^−1^, IV injection every 3 weeks during IV ipilimumab treatment period and 3 mg kg^−1^, IV injection every 2 weeks after IV ipilimumab treatment interruption. Treatment should be continued as long as clinical benefit is observed or until treatment is no longer tolerated by the patient, for a maximum of 12 months.

### Statistical methods

Safety and efficacy analysis were based on patients who took at least 1 dose of the study drug.

Primary end-point was the 6-month treatment related grade 3–4 toxicity event-free survival (EFS), defined as the time from inclusion to first documentation of treatment-related grade 3–4 toxicity. The number of patients to be included in the experimental arm was calculated considering a Fleming’s two-stage design and assuming the following, where *P* is the probability of success (tolerance of 6-month treatment), and α type 1 error rate of 10% and power of 90%: *P*_0_ = 50%, the 6-month treatment related grade 3–4 EFS below which the combination will considered too toxic; *P*_1_ = 70%, the 6-month treatment related grade 3–4 EFS above which the combination will be considered safe.

A total of 38 evaluable patients were required in the experimental arm. If the number of patients without treatment-related grade 3–4 adverse events after 6 months of treatment was <23/38, the conclusion of the design would be unacceptable tolerance. If the number of patients without treatment-related grade 3–4 adverse events after 6 months of treatment was ≥23/38, then the conclusion of the design would be acceptable tolerance.

No formal comparison between the two treatment arms was performed. The standard arm only serves as an internal control of the hypothesis of *P*_0_ and at least 19 patients were to be included.

Median follow-up was estimated with inverse Kaplan–Meier method. Overall survival was defined as the time from inclusion to documentation of death due to any cause. PFS was defined as the time from inclusion to documentation of tumour progression or death, whichever occurs first. PFS was be assessed by tumour measurements using RECIST 1.1. Overall survival and PFS was estimated using the Kaplan–Meier method and presented with Rothman’s 95% confidence intervals. The software used for statistical method analysis was SAS software version 9.4.

### Translational research

#### Nivolumab and Ipilimumab serum titrations

Nivolumab dosage method consists of one-day pre-analytical and analytical steps. For the pre-analytical step: Protein A purification was performed with Thermofisher scientific Protein A Spin plates; digestion was performed with Promega trypsin added to the samples and incubated overnight in a Thermomixer to thermostatically control the temperature under 300 rpm agitation; solid phase extraction was performed on Waters OASIS 96-well plates with washing steps with an aqueous solution and elution steps with an organic solvent; and sample concentration was performed with Speedvac vacuum concentrators with a temperature and vacuum level of 80 °C and 0.1 bar. For the analytical step: a Stepwave Xevo TQ-S UPLC-Mass Spectrometer (triple quadrupole, from Waters) with Masslynx software was used, over a range of 2.5–500 mg l^−1^. The nivolumab surrogate peptides used were ASGITFSNSGMHWVR (ASGI) and ASQSVSSYLAWYQQKPGQAPRLLIYDASNR (ASQS) with MRM transitions of 550.8 → 661.5 and 785.0 → 1,033.8, respectively. Bevacizumab was used as an internal standard with FTFSLDTSK (FTFS; MRM transition 523.4 → 797.4) and STAYLQMNSLR (STAY; MRM transition 642.3 → 861.3) as surrogate peptides. Separation was performed using a gradient of 0–1.0 min: 2.7% B, 1.0–6.0 min: 19% B, 6.0–6.10 min: 26.1% B, 6.1–8.0 min: 81% B, 8.10–10.0 min: 2.7% B.

Ipilimumab quantification was performed using an enzyme-linked immunosorbent assay (ELISA). Anti-ipilimumab Antibody Monovalent Fab, HCA330 (Bio-Rad AbD34433), was used as the capture antibody at a final concentration of 1 µg ml^−1^. The anti-ipilimumab antibody HCA329P (Bio-Rad AbD34429) was used as the detection antibody at 0.1 µg ml^−1^. Serum samples were diluted in PBS Tween 0.05% as follow: IV arm at baseline 1/200, IV arm after treatment at 1/40, IT arm at baseline 1/20, IT arm after treatment at 1/5. Enzymatic reaction was performed using QuantaBlu Fluorogenic Peroxidase Substrate Kit (Thermo Scientific). Fluorescence signal was quantified with Spectramax microplate reader (excitation 325 nm, emission 420 nm).

#### Tissue and blood sample analyses

Fresh whole blood was used to phenotype circulating immune cells by flow cytometry. Plasma was used to titre cytokines, chemokines and soluble factors. Only injected lesions were biopsied at baseline in the IT arm. Both injected and uninjected tumour lesions were biopsied at week 3 in patients from the IT arm. Up to six cores of tumour biopsies were collected from tumour lesions. WES and RNA-seq were performed on frozen tumour biopsies. IHC staining was performed on formalin-fixed paraffin-embedded (FFPE) tumour biopsies. Fresh tumour biopsies were also collected into media and sent immediately to a central laboratory for processing. Fresh tumour biopsies were subsequently mechanically and enzymatically dissociated and processed for flow cytometry. Cytokines, chemokines and soluble factors were titrated on supernatants (secretome) from fresh tumour biopsies. Tumour immunophenotyping and secretome analysis was performed for 22 and 17 patients, respectively. Sequential biopsies were performed at baseline (before treatment) and prior to cycle 2—that is, week 3 (on treatment)—in order to perform paired analysis whenever possible (*n* = 16). For patients in the IT arm, on treatment (week 3) biopsy samples were taken both from the injected and from an uninjected tumour (*n* = 12). Samples were collected for pathological analysis from 49 patients.

#### Immunohistochemistry

IHC was performed on 4-µm-thick, deparaffinized sections of FFPE tissue samples. The primary antibodies used in the study are listed in Extended Data Table 4; they were purchased from Abcam, Dako, DBioSystems, Dendritics, LS-Bio, MBL, R&D Systems, Roche Diagnostics and Spring Biosciences. Simple labelling immunoperoxidase techniques were used for CD26, CTLA4, HLA-I and HLA-II and mast cell tryptase. Three-plex chromogenic techniques were used for the co-detection of FOXP3/CD3/ICOS, CD8/PD-L1/Ki-67 and DC-Lamp/CD68/CD163. A four-plex chromogenic technique was used for the simultaneous detection of CD3/CD20/CD68/CD57. All techniques were performed on automated stainers (Benchmark ULTRA or Discovery, both from Ventana, or Bond RX, Leica Biosystems). The interpretation was performed by two experienced pathologists. The results were expressed according to the marker of interest, by H-scores, semi-quantitative evaluations or manual counting of labelled cells for the evaluation of cell densities (number of cells per mm²).

#### Flow cytometry, immune cell phenotyping and analysis from tumour biopsies

**Extended Data Fig. 10:**
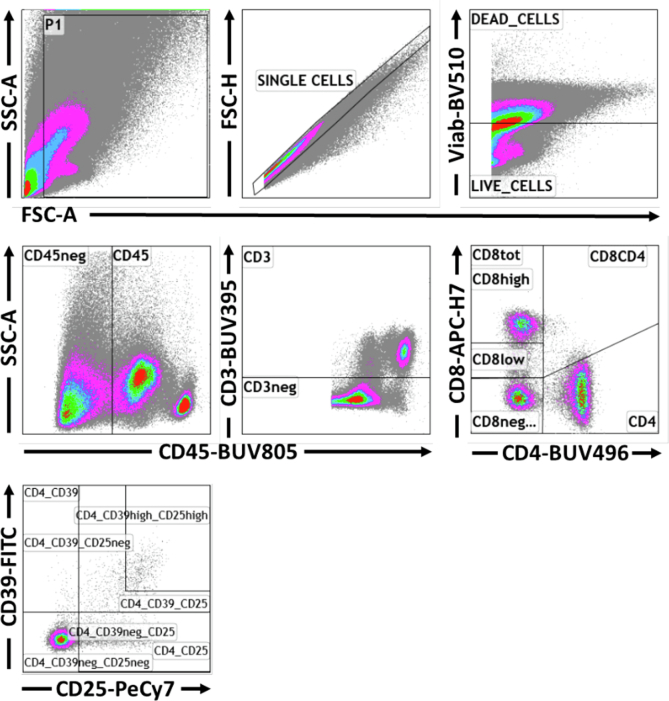
Gating strategy for flow cytometry analysis of fresh tumor biopsies.

Core biopsy samples from tumour at baseline and prior to cycle 2, week3, (injected and uninjected) were immediately placed into 1 ml of NaCl 0.9% and sent to the laboratory (LRTI–U1015). After a minimum of 30 min of incubation, fine-needle biopsies were mechanically dissociated with the bottom of a 2 ml syringe in a wet 70-µm filter placed at the top of a 50 ml centrifuge tube. Isolated cells were then washed by centrifugation and the pellet was re-suspended in an appropriate volume of NaCl 0.9% for the cell surface staining protocol. Antibodies mix (Extended Data Table 5) was composed of immune markers CD45, CD3, CD4, CD8 and HLA-ABC, activation markers HLA-DR and CD25, T_reg_ cell markers CD39 and CTLA4, immune checkpoint markers PD1, OX40 and TIGIT and a co-stimulator marker CD26. CTLA4 was first stained at 37 °C for 20 min before others surface antibodies were added and incubated at 4 °C for 15 min. Then cells were washed twice and acquisition were performed on an 18-colour flow cytometer BD Fortessa X20 (BD Biosciences). Data were processed in FCS 3.0 format and analysed with KALUZA software v2.1. From our population of interest, doublets were first excluded based on forward scatter height versus forward scatter area plot, and then viable cells were selected. Tumour-infiltrating T cells were then selected with a CD45^+^ followed by a CD3^+^ gate, and then divided into two sub-populations based on CD4 and CD8 expression (Extended Data Fig. 10).

#### Plasma and secretome cytokines measurement

Low-platelet plasma was generated on blood tubes collected in EDTA upon double high-speed centrifugation at 4 °C. Secretome experiments were standardized following the same procedure: one single 18 G fresh core needle biopsy was put into 1 ml saline an incubated at 4 °C for at least 30 min prior collection of the supernatants. To evaluate 51 soluble factors in patient plasma and/or 10 soluble factors in supernatant of biopsies, a Quickplex SQ120 platform enabling highly sensitive electro-chemo-luminescent detection (Meso Scale Discovery) was used following the manufacturer’s instructions. The analytes measured were GM-CSF; eotaxin and eotaxin-3; interleukins (IL-1α, IL-1β, IL-2, IL-4, IL-5, IL-6, IL-7, IL-8, IL-9, IL-10, IL-12p70, IL-12/IL-23p40, IL-13, IL-15, IL-16, IL-17A, IL-18, IL-18 BP, IL-21, IL-22, IL-27, IL-33, and receptor antagonist IL-1RA, CD25); interferons (IFNγ, IFNα2a, IFNλ (also known as IL-29)); IP-10; MIP1α and MIP1β; MDC; MCP1 and MCP4; TNFα and TNFβ; TARC; VEGF; fractalkine; calprotectin; immune checkpoint soluble proteins (PD1, PD-L1 and CTLA4); enzymatic proteins (GZMA and GZMB); BCA1; and TGFβ1, TGFβ2 and TGFβ3. Absolute concentrations of soluble analytes (in pg ml^−1^ or fg ml^−1^) in patient samples were calculated by use of a four-point-fit calibration curve of the standard dilutions (MSD Discovery Workbench analysis software) and were considered detectable if both runs of each sample had a signal greater than the analyte- and plate-specific lower limit of detection.

#### Flow cytometry immune cells phenotyping and analysis from blood samples

Immune cell phenotyping from blood samples was performed according to a previously described methodology^49^. The panels used are summarized in the Extended Data Table 6.

#### WES and RNA analysis

The exome libraries were prepared using the Agilent V6 XT kit and sequenced on a BGISEQ-500 instrument at BGI according to the manufacturer’s protocols.

Reads were mapped to the GRCh37 human reference genome using BWA-MEM (v0.7.12) software. A standard GATK best practice pipeline was used to process the samples and call somatic genetic variants using GATK Mutect2^50^. Variants were annotated with oncotator software (v1.9.9.0) and oncoKB database. SCNA calling was done with FACETS software (v 0.5.14). All processing steps were implemented with a snakemake pipeline (v5.4.0)^51^. Quality control of FASTQ and BAM files was performed with FASTQC (v0.11.7) and samtools (v1.9) respectively^52^. Only somatic variants with PASS flag and supported by minimum three reads with at least one read from each strand were used for the further analysis. Known mutational signatures for WES mutations were deconvoluted using R package MutationalPatterns (fit_to_signatures_strict(), max_delta = 0.003) based on the mutational matrices from SigProfilerMatrixGenerator. Oncoplot was constructed using maftools R package^53,54^.

RNA samples were sequenced on BGISEQ-500 instrument. The reads were pseudoaligned on the human transcriptome database (hg38) with the Kallisto pipeline and final TPM values for each gene in each sample were received as described^55^. TPM values were used to perform absolute deconvolution of the tumour microenviroment using Kassandra algorithm (tumour mode)^56^. SCNA genomic instability score was established as follows: for each sample, the absolute copy number (ACN) profile generated by ASCAT through EaCoN was used. For each chromosome taken independently, the basal ACN level was identified as the one with the longest total width. Then, for each chromosome, ACN levels were converted into the absolute difference to this basal level [aDCN = (if the basis was 3 copies, a 1 copy segment value will be 2; 2 copies ≥ 1; 3 copies ≥ 0; 4 copies ≥ 1; etc.)]. The final SCNA score was computed as the width-ponderated sum of each of these converted copy number-to-basis values, divided by the total covered genome length.

#### Statistical methods

The data from IHC, flow cytometry (tumour biopsies and blood), secretome and plasma were processed with the dplyr package (1.1.4) for later use.

A paired two-sided Wilcoxon test was used to analyse statistical differences between baseline and week 3. A Wilcoxon test was used for statistical analysis between two conditions/groups (IT versus IV or no DCB versus DCB) at baseline. Statistical analysis was performed using the rstatix package (0.7.2) with the wilcox_test function, with the parameter paired = TRUE for paired analysis. Paired box plots and box plots were generated using the packages ggpubr (0.6.0) and ggplot2 (3.4.4). While the paired Wilcoxon analysis used only patients with a value between the two time points, the visual representation used all patients, even those not included in the Wilcoxon test. Centre values were defined as medians, consistent with the use of a non-parametric Wilcoxon test. All these analyses were performed using R software (v.4.3.3).

### Reporting summary

Further information on research design is available in the Nature Portfolio Reporting Summary linked to this article.

## Online content

Any methods, additional references, Nature Portfolio reporting summaries, source data, extended data, supplementary information, acknowledgements, peer review information; details of author contributions and competing interests; and statements of data and code availability are available at 10.1038/s41586-026-10341-w.

## Supplementary information

Reporting Summary

Peer Review File

## Figures and Tables

**Extended Data Table 1:**
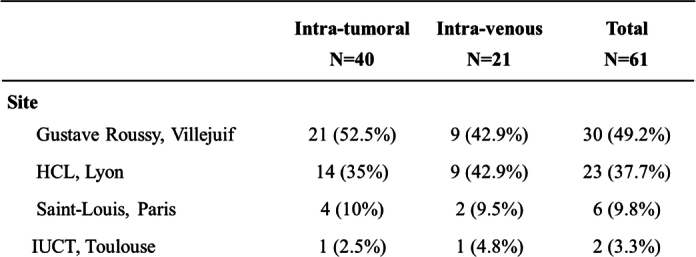
Patient enrollment per treatment arm and per investigational site

Gustave Roussy: Institut Gustave Roussy (Comprehensive Cancer Center); HCL: Hospices Civils de Lyon (University Hospital); Saint-Louis: Assistance-Publique Hôpitaux de Paris, Hôpital Saint-Louis (University Hospital); IUCT: Institut Universitaire de Cancérologie de Toulouse (joint venture between Comprehensive Cancer Center & University Hospital).

**Extended Data Table 2:**
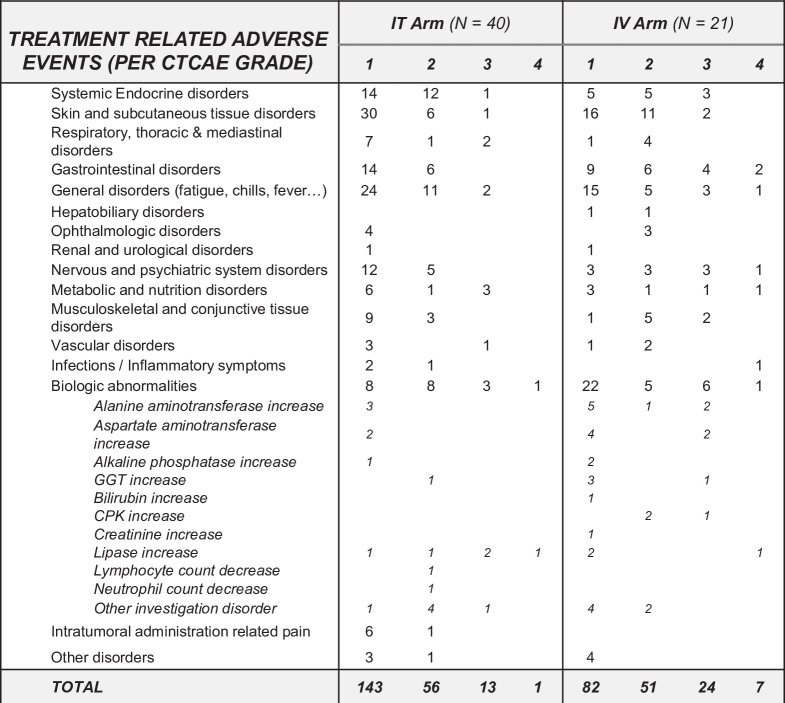
List of the most frequent treatment-related adverse events according to CTCAE v.4.0 grades and per treatment arm

**Extended Data Table 3:**
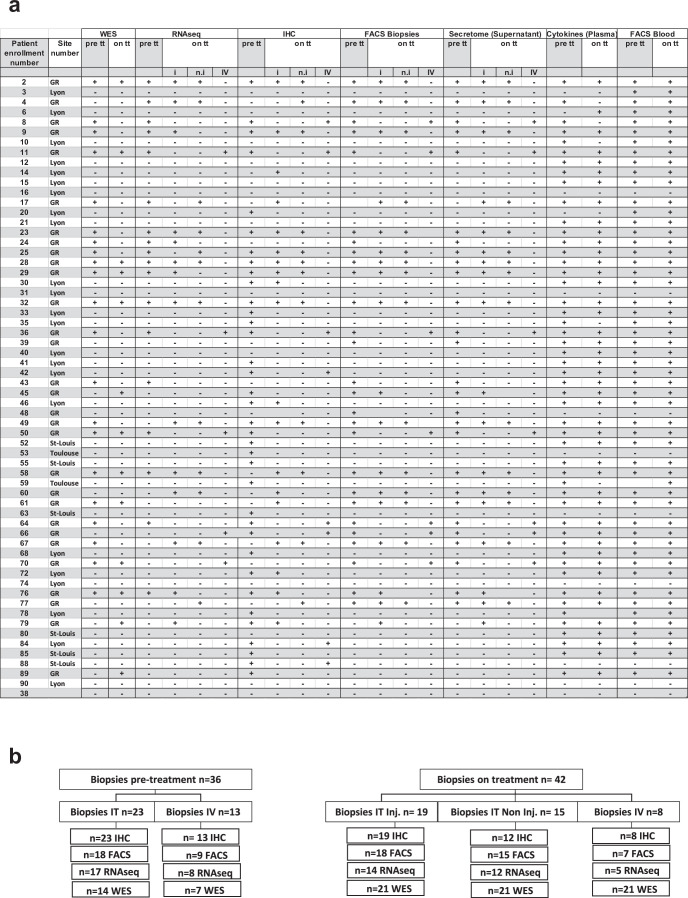
**a**. Sample Manifest detailing which samples were available for what patients. **b**. Sample Distribution between Therapeutic Arms

**Extended Data Table 4:**
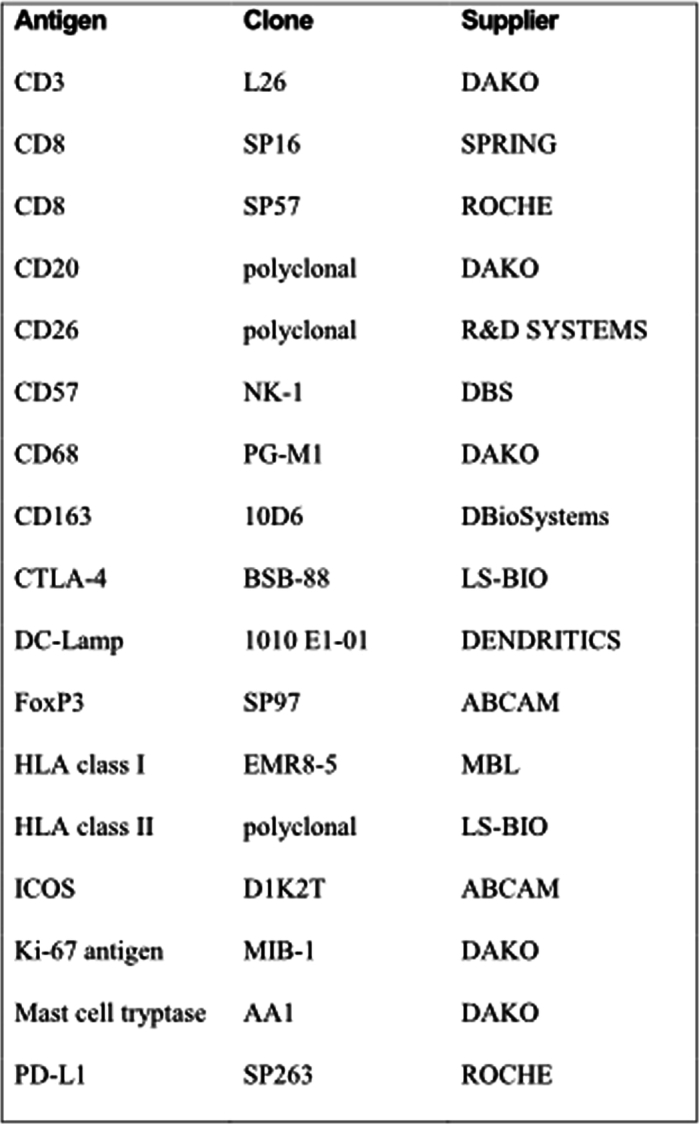
List of primary antibodies used for Immunohistochemistry

**Extended Data Table 5:**
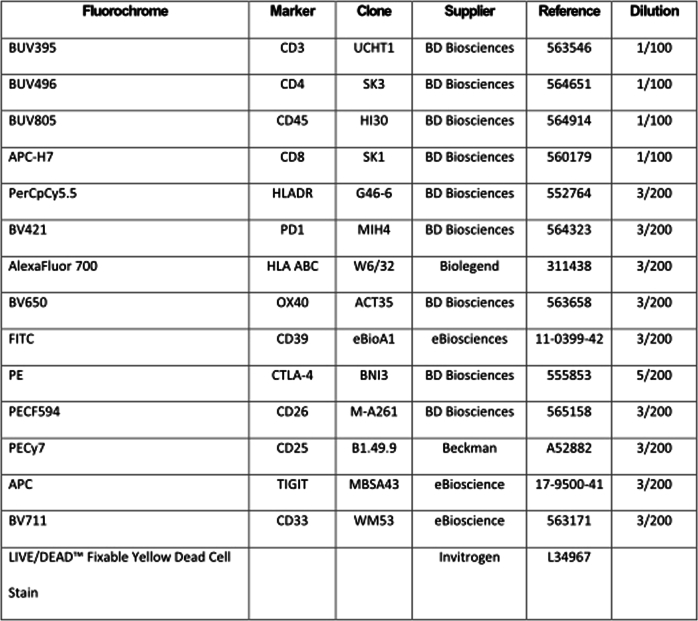
List of antibodies and fluorochromes used for tumor biopsies phenotyping

**Extended Data Table 6:**
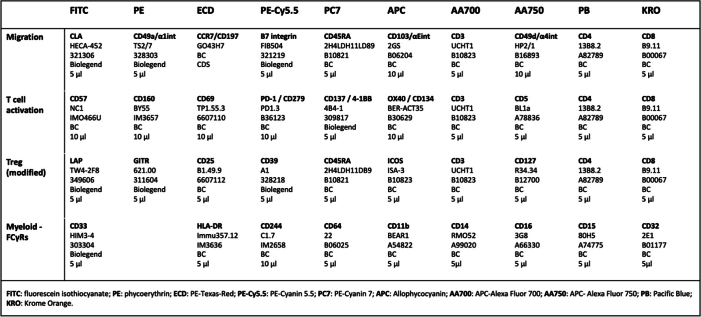
List of antibodies and fluorochromes used for blood cells phenotyping

**Extended Data Table 7:**
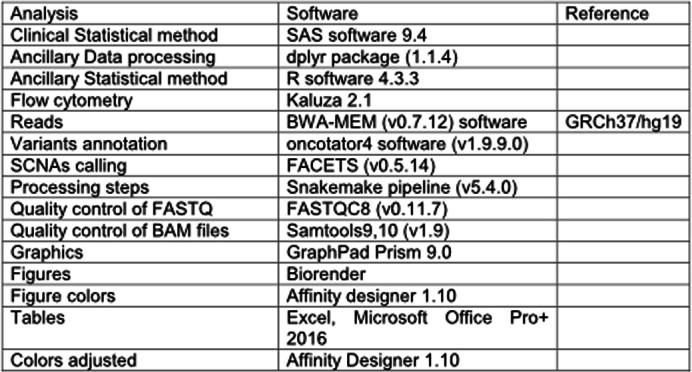
Software Licenses

## Data Availability

Owing to ethical and legal restrictions related to the protection of human participant privacy, the clinical datasets generated and analysed during this study are not publicly available. Within 6 months of publication, anonymized individual participant data, the annotated case report form, study protocol, reporting and analysis plan, dataset specifications, raw dataset, analysis-ready dataset and the clinical study report will be made available for research proposals approved by an internal review committee at Gustave Roussy. Requests for data access should be submitted to transfert@gustaveroussy.fr. Access will be granted following approval of the research proposal and execution of a data access agreement. Software licenses are listed in Extended Data Table 7.
