# Supplementary material for: Safety and efficacy of intratumoural anti-CTLA4 with intravenous anti-PD1
Source: Nature. 2026 Apr 29;655(8121):219–29. doi: 10.1038/s41586-026-10341-w (PMC13323097; doi:10.1038/s41586-026-10341-w)
Supplement: Supplementary file 1 — Reporting Summary [file 41586_2026_10341_MOESM1_ESM.pdf]

nature portfolio

Corresponding author(s): Aurélien Marabelle

Last updated by author(s): 22 Dec 2025

# Reporting Summary

Nature Portfolio wishes to improve the reproducibility of the work that we publish. This form provides structure for consistency and transparency in reporting. For further information on Nature Portfolio policies, see our Editorial Policies and the Editorial Policy Checklist.

## Statistics

For all statistical analyses, confirm that the following items are present in the figure legend, table legend, main text, or Methods section.

| n/a | Confirmed | |
|---|---|---|
| ☐ | ☒ | The exact sample size (*n*) for each experimental group/condition, given as a discrete number and unit of measurement |
| ☐ | ☒ | A statement on whether measurements were taken from distinct samples or whether the same sample was measured repeatedly |
| ☐ | ☒ | The statistical test(s) used AND whether they are one- or two-sided<br>*Only common tests should be described solely by name; describe more complex techniques in the Methods section.* |
| ☐ | ☒ | A description of all covariates tested |
| ☐ | ☒ | A description of any assumptions or corrections, such as tests of normality and adjustment for multiple comparisons |
| ☐ | ☒ | A full description of the statistical parameters including central tendency (e.g. means) or other basic estimates (e.g. regression coefficient) AND variation (e.g. standard deviation) or associated estimates of uncertainty (e.g. confidence intervals) |
| ☐ | ☒ | For null hypothesis testing, the test statistic (e.g. *F*, *t*, *r*) with confidence intervals, effect sizes, degrees of freedom and *P* value noted<br>*Give P values as exact values whenever suitable.* |
| ☒ | ☐ | For Bayesian analysis, information on the choice of priors and Markov chain Monte Carlo settings |
| ☒ | ☐ | For hierarchical and complex designs, identification of the appropriate level for tests and full reporting of outcomes |
| ☒ | ☐ | Estimates of effect sizes (e.g. Cohen's *d*, Pearson's *r*), indicating how they were calculated |

*Our web collection on statistics for biologists contains articles on many of the points above.*

## Software and code

Policy information about availability of computer code

| | |
|---|---|
| Data collection | The eCRF of the trial was made sith the software InferMed Macro 4.0. |
| Data analysis | Below the listing of software used for data analysis:<br>Clinical Statistical method :SAS software 9.4<br>Ancillary Data processing : dplyr package (1.1.4)<br>Ancillary Statistical method : R software 4.3.3<br>Flow cytometry : Kaluza 2.1<br>Reads : BWA-MEM (v0.7.12) software GRCh37/hg19<br>Variants annotation : oncotator4 software (v1.9.9.0)<br>SCNAs calling :FACETS (v0.5.14)<br>Processing steps : Snakemake pipeline (v5.4.0)<br>Quality control of FASTQ : FASTQC8 (v0.11.7)<br>Quality control of BAM files : Samtools9,10 (v1.9)<br>Graphics : GraphPad Prism 9.0<br>Figures : Biorender<br>Figure colors : Affinity designer 1.10 |

For manuscripts utilizing custom algorithms or software that are central to the research but not yet described in published literature, software must be made available to editors and reviewers. We strongly encourage code deposition in a community repository (e.g. GitHub). See the Nature Portfolio guidelines for submitting code & software for further information.

## Data

Policy information about availability of data

All manuscripts must include a data availability statement. This statement should provide the following information, where applicable:

- Accession codes, unique identifiers, or web links for publicly available datasets
- A description of any restrictions on data availability
- For clinical datasets or third party data, please ensure that the statement adheres to our policy

Due to ethical and legal restrictions related to the protection of human participant privacy, the clinical datasets generated and analyzed during this study are not publicly available. Within 6 months of publication, anonymized individual participant data, the annotated case report form, study protocol, reporting and analysis plan, dataset specifications, raw dataset, analysis-ready dataset, and the clinical study report will be made available for research proposals approved by an internal review committee at Gustave Roussy.Requests for data access should be submitted to transfert@gustaveroussy.fr. Access will be granted following approval of the research proposal and execution of a data access agreement.

## Research involving human participants, their data, or biological material

Policy information about studies with human participants or human data. See also policy information about sex, gender (identity/presentation), and sexual orientation and race, ethnicity and racism.

| | |
|---|---|
| Reporting on sex and gender | There was no restriction on sex and gender in the eligibility criteria of the protocol and therefore on the patients enrolled in the trial |
| Reporting on race, ethnicity, or other socially relevant groupings | There was no restriction on race, ethnicity or social group in the eligibility criteria of the protocol and therefore in the patients enrolled in the trial. |
| Population characteristics | The study included 63 untreated patients with unresectable metastatic mélanoma who were eligible to receive first-line double immunotherapy, 61 participants started the treatment. The cohort comprised 62% male and 38% female patients. The mean age at randomization was 55 years. 49% percent of patients were treated at Gustave Roussy  PD-L1 status was positive in 16% of patients. BRAF mutational status was wild-type in 56% of patients. |
| Recruitment | Participants were enrolled in the randomized multicenter Phase 1b NIVIPIT trial (NCT02857569). Patients were randomly assigned in a 2:1 ratio to receive intravenous nivolumab (1 mg/kg) combined with either intratumoral ipilimumab (0.3 mg/kg) or intravenous ipilimumab (3 mg/kg). |
| Ethics oversight | This study was an investigator initiated trial sponsored by Gustave Roussy and covered by a Biomedical Research Promoter Civil Liability insurance contract (contract #124.895) in accordance with the provisions of the French law (Decree 2006-477 of 26 April 2006 and Article L.1121-10 of the French Public Health Code). This study was approved by the French Health Agency (Agence Nationale de Sécurité du Médicament) on March 29th 2016 (ANSM #160104A-12) and by the national ethics committee (Comité de Protection des Personnes Ile-de-France VIII) on Feb 16th 2016 (CPP #160215). The study was registered on EUDRACT (2015-005429-37) on Nov 30th 2015 and on clinicaltrials.gov (NCT02857569) on July 19th 2016. The study was conducted in accordance with the Declaration of Helsinki and international Conference on Harmonization Good Clinical Practice (GCP). All investigators were GCP certified. The study was approved by the Gustave Roussy institutional review board (CSET 2015/2334) on Nov 24th 2015. All patients provided written informed consent prior enrollment in the trial and before any study specific procedure for clinical data and ancillary analysis anonymous use. Ipilimumab and nivolumab were supplied by Bristol-Myers Squibb (BMS). |

Note that full information on the approval of the study protocol must also be provided in the manuscript.

# Field-specific reporting

Please select the one below that is the best fit for your research. If you are not sure, read the appropriate sections before making your selection.

☒ Life sciences ☐ Behavioural & social sciences ☐ Ecological, evolutionary & environmental sciences

For a reference copy of the document with all sections, see nature.com/documents/nr-reporting-summary-flat.pdf

# Life sciences study design

All studies must disclose on these points even when the disclosure is negative.

| | |
|---|---|
| Sample size | The number of patients to be included in the experimental arm was calculated considering a Flemming's two-stage design and assuming the following, where ? is the true probability of success (tolerance of six-month treatment):<br>p0 = 50% : the tolerance, defined as treatment related grade 3-4 event-free survival at 6 months below which the combination will be considered as too toxic<br>p1 = 70% : the tolerance, defined as treatment related grade 3-4 event-free survival at 6 months above which the combination will be considered as safe.<br>Alpha type I error rate = 0.10 and a power of 90%<br>A total of 38 evaluable patients is required in the experimental arm. |

No formal comparison between the experimental arm and the standard arm (Arm B) will be performed. The standard arm will only be used as an internal control of the hypothesis on ⍰0 to interpret the results obtained in the experimental arm (randomization avoids patient selection biases). Considering the control arm's role, a 2:1 randomisation ratio is envisaged, thus 19 patients will be enrolled in the standard arm.
To take into account patients non evaluable for tolerance (ex: patients stopping treatment for ineficacy), a total sample size of 65 randomized patients is required for this Phase I/II trial.

**Data exclusions**

There were no data exclusions. Two patients were randomized but did not receive experimental treatment and consequently, they were considered as not evaluable for primary endpoint.

**Replication**

40 patients were treated in the experimental arm. 21 patients were treated in the control arm

**Randomization**

After the subject's initial eligibility is established and informed consent has been obtained, the subject must be enrolled into the study and a subject number should be obtained from the sponsor. The registration will be performed before any specific study procedure including the biopsy. Every subject that signs the informed consent form must be assigned a subject number. The investigator or designee will register the subject for enrollment by following the enrollment procedures established by the sponsor. The following information is required for enrollment:
- Date that informed consent was obtained
- Date of birth
- Gender at birth
Once enrolled, subjects that have met all eligibility criteria will be ready to be randomized through. The following information is required for subject randomization:
- Subject number
- Date of birth
- PD-L1 status (PD-L1 positive vs. PD-L1 negative/indeterminate)
- BRAF status
- M Stage
For subjects meeting all eligibility criteria, treatment will be randomized in a 2:1 ratio to the 2 arms (2 patients in the ipilimumab IT arm for 1 patient in the IV ipilimumab arm) stratified by the following factors:
- PD-L1 status
PD-L1 positive (⍰ 5% tumor cell membrane staining in a minimum of a hundred evaluable tumor cells) vs
PD-L1 negative (< 5% tumor cell membrane staining in a minimum of a hundred evaluable tumor cells)/ PD-L1 indeterminate (tumor cell membrane scoring hampered by high cytoplasmic staining or melanin content)
- M Stage (see Appendix 4)
M0/M1a/M1b vs M1c
- BRAF status
Wild type vs Mutated
Blocked Randomization with Randomly Selected Block Sizes will be used to generate 8 lists of treatment allocation corresponding to the 8 possible combinations of the three factors for randomization. The SAS 9.4 Proc plan procedure will be used.
A second form "Randomisation" will have to be sent for the randomization of the patient using the same procedures. The confirmation of randomisation will be automatically sent to the treating centre, including the treatment allocated by randomization. The first treatment dose has to be administered within 7 days following randomization.

**Blinding**

This was an open trial, no relevancy of blinding the treatment allocation because of the different route of administration (IT or IV) of the tested drugs.

# Reporting for specific materials, systems and methods

We require information from authors about some types of materials, experimental systems and methods used in many studies. Here, indicate whether each material, system or method listed is relevant to your study. If you are not sure if a list item applies to your research, read the appropriate section before selecting a response.

## Materials & experimental systems

| n/a | Involved in the study | |
|---|---|---|
| ☐ | ☒ | Antibodies |
| ☒ | ☐ | Eukaryotic cell lines |
| ☒ | ☐ | Palaeontology and archaeology |
| ☒ | ☐ | Animals and other organisms |
| ☐ | ☒ | Clinical data |
| ☒ | ☐ | Dual use research of concern |
| ☒ | ☐ | Plants |

## Methods

| n/a | Involved in the study | |
|---|---|---|
| ☒ | ☐ | ChIP-seq |
| ☐ | ☒ | Flow cytometry |
| ☒ | ☐ | MRI-based neuroimaging |

## Antibodies

**Antibodies used**

cf manuscript

**Validation**

All primary antibodies used in this study were validated for both the human specie and the flow cytometry application reported. For each antibody, validation information was obtained from the manufacturer's datasheet, supplemented by citations from peer-reviewed publications and antibody profiles available in online databases.

For the antibody BD Horizon™ BUV395 Mouse Anti-Human CD3 (Ref 563546), the manufacturer states that it is validated for human and Flow Cytometry application. This antibody was previously published in Structural analysis of cancer-relevant TCR-CD3 and peptide-MHC complexes by cryoEM. (Nature Communications, April 2023 by Saotome, K., Dudgeon, D., et al.)
For the antibody BD Horizon™ BUV496 Mouse Anti-Human CD4 (Ref 612936), validation by the manufacturer includes an Isotype control on PBMCs, Human QC tests, flow cytometry experiments routinely tested, and prior use reported in the publication called Antitumor immunity induced by antibody-based natural killer cell engager therapeutics armed with not-alpha IL-2 variant (Cell Reports Medicine on 18 October 2022 by Demaria, O., Gauthier, L., et al.).
All antibody validation details, including manufacturer statements and literature support are summarize in internet links in the following table

Fluorochrome Marker Internet link
BUV395 CD3 https://www.bdbiosciences.com/en-us/products/reagents/flow-cytometry-reagents/research-reagents/single-color-antibodies-ruo/buv395-mouse-anti-human-cd3.563546?tab=product_details

BUV496 CD4 https://www.bdbiosciences.com/en-us/products/reagents/flow-cytometry-reagents/research-reagents/single-color-antibodies-ruo/buv496-mouse-anti-human-cd4.612936?tab=product_details

BUV805 CD45 https://www.bdbiosciences.com/en-us/products/reagents/flow-cytometry-reagents/research-reagents/single-color-antibodies-ruo/buv805-mouse-anti-human-cd45.612891?tab=product_details

APC-H7 CD8 https://www.bdbiosciences.com/en-us/products/reagents/flow-cytometry-reagents/research-reagents/single-color-antibodies-ruo/apc-h7-mouse-anti-human-cd8.560179?tab=product_details

PerCpCy5.5 HLADR https://www.bdbiosciences.com/en-us/products/reagents/flow-cytometry-reagents/research-reagents/single-color-antibodies-ruo/percp-cy-5-5-mouse-anti-human-hla-dr.552764?tab=product_details

BV421 PD-1 https://www.bdbiosciences.com/en-us/products/reagents/flow-cytometry-reagents/research-reagents/single-color-antibodies-ruo/bv421-mouse-anti-human-cd279-pd-1.564323?tab=product_details

AlexaFluor 700 HLA ABC https://www.biolegend.com/en-us/products/alexa-fluor-700-anti-human-hla-a-b-c-antibody-12389

BV650 OX40 https://www.bdbiosciences.com/en-us/products/reagents/flow-cytometry-reagents/research-reagents/single-color-antibodies-ruo/bv650-mouse-anti-human-cd134.563658?tab=product_details

FITC CD39 https://www.thermofisher.com/antibody/product/CD39-Antibody-clone-eBioA1-A1-Monoclonal/11-0399-42

PE CTLA-4 https://www.bdbiosciences.com/en-us/products/reagents/flow-cytometry-reagents/research-reagents/single-color-antibodies-ruo/pe-mouse-anti-human-cd152.555853?tab=product_details

PE-CF594 CD26 https://www.bdbiosciences.com/en-us/products/reagents/flow-cytometry-reagents/research-reagents/single-color-antibodies-ruo/pe-cf594-mouse-anti-human-cd26.565158?tab=product_details

PECy7 CD25 https://www.beckman.fr/reagents/coulter-flow-cytometry/antibodies-and-kits/single-color-antibodies/cd25/a52882

APC TIGIT https://www.thermofisher.com/antibody/product/TIGIT-Antibody-clone-MBSA43-Monoclonal/17-9500-41

LIVE/DEAD™ Fixable Yellow Dead Cell Stain https://www.thermofisher.com/order/catalog/product/L34967?SID=srch-hj-L34967

## Clinical data

Policy information about clinical studies
All manuscripts should comply with the ICMJE guidelines for publication of clinical research and a completed CONSORT checklist must be included with all submissions.

Clinical trial registration: NCT02857569 (https://clinicaltrials.gov/study/NCT02857569?term=NCT02857569%20&rank=1)

Study protocol: The full trial protocol can be assessed in the supplementary Files

Data collection: First Patient In (FPI) on September 30th 2016. Last PAtietn In (LPI) July 18th 2019. Last Patient Last Visit (LPLV : July 30th 2022

Outcomes: The primary endpoint is the 6 month treatment-related grade 3-4 toxicity event-free survival (EFS).

Adverse Events (AEs) will be graded by the investigator according to the Common Terminology Criteria for Adverse Events (CTCAE) version 4.0 and coded using the Medical Dictionary for Regulatory Activities (MedDRA). Treatment-related grade 3-4 toxicity event-free survival free survival (EFS) is defined as the time from randomization to first documentation of treatment-related grade 3-4 toxicity or to death, whichever occurs first. Any patient that discontinues due to a reason other than toxicity (for example, progression, patient/investigator decision) will have their time-to-event data either censored at the date of discontinuation, if known, or at the follow-up visit if no treatment-related grade 3-4 toxicity is found during this visit.

The secondary endpoints are :
- To further explore the types of toxicities generated by the combination
therapy in the two arms.
- To evaluate the efficacy of IT ipilimumab in combination with IV

nivolumab.
- To identify early imaging markers of response.
-To identify predictive biomarkers of response
-To demonstrate the lower systemic exposure to Ipilimumab in the IT arm (PK data)
- To assess response to treatement using several endpoints for efficacy based either on RECIST1.1, or on immune-related Response Criteria (irRC) or on irRECIST

To determine the frequence and severity of immune related adverse events (irAEs), notably beyond 6 months. To establish the Objective Response Rate (ORR) at 12 month, the 12 months Progression Free Survival (PFS), the 12 months Overall Survival (OS), the 24 months PFS and the 24 months OS. To evaluate the predictive value of :
i) early tumor vascularization changes by DCE-US
ii) early metabolic changes by PET-CT
To perform a translational scientific study with the subsequent aims :
i) Identify predictive biomarkers of tumor response
ii) Determine which immune checkpoints are upregulated upon ipilimumab+nivolumab therapy within tumors of primary and secondary resistant patients
iii) Sequence BCR & TCR of tumor infiltrative B and T-cells expanding upon ipilimumab+nivolumab therapy in responding patients

## Plants

Seed stocks

*Report on the source of all seed stocks or other plant material used. If applicable, state the seed stock centre and catalogue number. If plant specimens were collected from the field, describe the collection location, date and sampling procedures.*

Novel plant genotypes

*Describe the methods by which all novel plant genotypes were produced. This includes those generated by transgenic approaches, gene editing, chemical/radiation-based mutagenesis and hybridization. For transgenic lines, describe the transformation method, the number of independent lines analyzed and the generation upon which experiments were performed. For gene-edited lines, describe the editor used, the endogenous sequence targeted for editing, the targeting guide RNA sequence (if applicable) and how the editor was applied.*

Authentication

*Describe any authentication procedures for each seed stock used or novel genotype generated. Describe any experiments used to assess the effect of a mutation and, where applicable, how potential secondary effects (e.g. second site T-DNA insertions, mosiacism, off-target gene editing) were examined.*

## Flow Cytometry

### Plots

Confirm that:

☒ The axis labels state the marker and fluorochrome used (e.g. CD4-FITC).

☒ The axis scales are clearly visible. Include numbers along axes only for bottom left plot of group (a 'group' is an analysis of identical markers).

☒ All plots are contour plots with outliers or pseudocolor plots.

☒ A numerical value for number of cells or percentage (with statistics) is provided.

### Methodology

Sample preparation

- Tumor biopsies samples
Core biopsy samples from tumor at baseline and prior to cycle 2, week3, (injected and non-injected) were immediately placed into 1ml of NaCl 0.9% and sent to the laboratory (LRTI – U1015). After a minimum of 30 minutes of incubation, fine-needle biopsies were mechanically dissociated with the bottom of a 2ml syringe in a wet 70µm filter placed at the top of a 50ml centrifuge tube. Isolated cells were then washed by centrifugation and the pellet was re-suspended in an appropriate volume of NaCl 0.9% for cell surface staining protocol.

- Blood samples
Antibody staining
Whole blood (100 µL) sampled with anticoagulant was mixed with 10 µL of Perfix-NC R1 buffer, vortexed immediately for 2-3 seconds and incubated for 15 min at room temperature in the dark. 600 µl of Perfix-NC R2 buffer were added, 355 µl were transferred in the Duraclone tube and liquid antibodies were added. After vortexing, tubes were incubated for 60 min at room temperature in the dark. PBS 1X (3 mL) was added to the tubes, incubated for 5 min at room temperature in the dark before centrifugation for 6 min at 250g. The supernatant was removed to leave the pellet dried and the cells were resuspended in 3 mL of 1X Perfix-NC R3 buffer prior to another 6-min centrifugation at 250g. The pellet was dried and resuspended in 300 µL of 1X R3 buffer. Tubes were protected from light and stored at 4°C until the acquisition on a cytometer within the next 24 h.
For all panels, all conjugated antibodies were dried in Duraclone tubes except for the “Treg modified panel” and liquid antibodies. Versalyse was purchased from Beckman coulter. For each panel, 100 µl of whole blood were added in the corresponding Duraclone and the liquid antibodies were added when necessary. After vortex, tubes were incubated for 15

min at room temperature in the dark. 2 ml of Versalyse containing 50 µl of fixative solution were then added prior to another incubation of 15 min at room temperature in the dark. Cells were centrifuged 6 min at 250g, resuspended in 3 ml of 1X PBS and centrifuged again. The pellet was finally resuspended in 250 µl of 1X PBS and stored at 4°C without light until the acquisition on a cytometer within the next 24h.

| | |
|---|---|
| Instrument | Flow cytometer used for cells from tumor biopsies: BD Fortessa X20 (BD Biosciences)<br>Flow cytometer used for cells from blood: Gallios (Beckman Coulter) |
| Software | KALUZA software 2.1 |
| Cell population abundance | Cells from tumor biopsies: Cell population abundance<br>During the clinical trial all biopsies were collected using a 18 gauge (18G) Chiba needles with a single endhole to avoid size variability between patients. In this study, as the graph below shows, dissociated biopsies are composed of a number of live cells going from 2105 to 4337736 and a proportion of immune infiltrating cells varing from 0,01% to 82,4%. Regarding the proportion of T-Regs like cells expressing CD4+CD25highCD39high, it goes from 0% to 4.87% among live immune cells.<br>We can notice that 29 biopsies over 51 are composed by less than 200000 live cells. |
| Gating strategy | From our population of interest, doublets were first excluded based on forward-scatter-Height versus forward-scatter Area plot. Then live cells were selected from the negative gate of LIVE/DEAD™ Fixable Yellow Dead Cell Staining. Tumor infiltrating T-lymphocytes were then selected with a CD45+ followed by a CD3+ gate, and then were divided into two sub-populations based on CD4 and CD8 expression. From CD4-positive and CD8-negative gate, T-Regs like populations were define by CD25 and CD39 high-expression |

☒ Tick this box to confirm that a figure exemplifying the gating strategy is provided in the Supplementary Information.
