# Supplementary material for: Safety and efficacy of intratumoural anti-CTLA4 with intravenous anti-PD1
Source: Nature. 2026 Apr 29;655(8121):219–29. doi: 10.1038/s41586-026-10341-w (PMC13323097; doi:10.1038/s41586-026-10341-w)
Supplement: Supplementary file 2 — Peer Review File [file 41586_2026_10341_MOESM2_ESM.pdf]

nature portfolio

Peer Review File

Safety and Efficacy of Intratumoral Anti-CTLA4 with Intravenous Anti-PD1

Corresponding Author: Professor Aurelien Marabelle

Any redactions in this file are there to maintain patient confidentiality, the confidentiality of unpublished data, or to remove third-party material.

This file contains all reviewer reports in order by version, followed by all author rebuttals in order by version.

Version 0:

Reviewer comments:

Referee #1

(Remarks to the Author)
This manuscript reports the results of a randomized Phase 1b trial (NCT02857569), evaluating the safety and efficacy of intratumoral (IT) ipilimumab (0.3 mg/kg) combined with intravenous (IV) nivolumab (1 mg/kg), compared to the standard IV ipilimumab (3 mg/kg) plus nivolumab, in treatment-naive patients with advanced melanoma. The study met its primary endpoint, showing reduced grade ≥3 irAEs in the IT arm (22.6%) compared to the IV arm (57.1%). The manuscript also presents an extensive translational program, identifying predictors of durable clinical benefit (DCB), notably the presence of intratumoral activated Tregs and M2 macrophages.

General Comments
The study addresses a critical clinical issue: reducing anti-CTLA4-related toxicities while maintaining efficacy. Intratumoral delivery of ipilimumab is innovative and potentially practice-changing. The study showed a marked reduction in systemic toxicity and extensive translational data set (biopsies, secretome, flow cytometry).

Efficacy comparison underpowered but informative. While not powered for efficacy, the IT arm showed a 50% response in non-injected lesions, close to the 62% in the IV arm. It would be good to stratify responses by injection site or lesion type, if possible.

Unexpectedly, Tregs and M2 macrophages predicted response (DCB). This highlights the complexity of tumor immune ecosystems. The CD25+CD39+CD4+ Treg characterization via flow cytometry is robust; please, discuss feasibility of using this as a clinical biomarker.

Pharmacokinetics show lower ipilimumab levels in the IT arm. Nivolumab levels were paradoxically lower in the IV ipilimumab group. This finding adds mechanistic insight into immune activation versus toxicity.

Minor Comments
Abstract: Consider clarifying the sentence on cross-reactivity (line 9).
Figures: Consider summarizing safety/efficacy trade-offs in a new schematic figure.
Extended Data: Move key Treg depletion figure (Ext Data Fig 6) to main figures.
Typo in abstract: “those hypothesis” → “these hypotheses”.

Referee #2

(Remarks to the Author)
The manuscript “Safety and Efficacy of Intratumoral Anti-CTLA4 with Intravenous Anti-PD1” by Tselikas et al. is a very impressive body of work describing a timely study in the exciting field of intratumoral immunotherapy. It shows the safety and clinical efficacy of i.t. anti-CTLA4 and i.v. anti-PD-1 in advanced melanoma to be equivalent to i.v. Although a non-significant advantage in terms of survival was found in te i,v, arm.

We are very excited about this manuscript. The described study is highly original and innovative and we and strongly feel that it should be published to the benefit of the cancer immunotherapy field. Lower irAE rates combined with optimal efficacy would facilitate wider application of this powerful dual immunotherapy, particular in earlier disease stages and the neoadjuvant setting.

Overall, the approach is valid and the quality of the presented data is high. The used statistics are appropriate. Overall the conclusions are robust and justified. Nevertheless, some statements are an over-interpretation; we have some comments and feedback to further improve the quality of the work and help showcase the very important results of this study more effectively, see below.

Major comments:

In the immunomonitoring section, we think more emphasis should be placed on the comparison between intratumoral and intravenous administration, as this was the aim of the trial and paper. The fact that no, or very little difference can be found in immune response between the two different strategies is of profound interest, as this proves that intratumoral delivery of anti-CTLA4 equally and similarly effects immune activation as intravenous administration.

We miss the likely role of the tumor-draining lymph nodes in the efficacy of intratumoral injection in the discussion of the manuscript. The authors discuss that the they have two hypotheses for the clinical responses observed in the non-injected lesions; one is optimal exposure of the TME (but what about TDLN?) The other that the intratumorally injected dose leaks into the periphery, and they use a reference to back this claim. However, in that referenced publication, a similar dose was injected systemically and intratumorally, whereas in this study a tenfold lower dose was used intratumorally. As such this hypothesis is not backed by the listed reference. In another study of locally injected tremelimumab (van Pul et al. Sci Immunol 7: eabn8097 2022) dose levels similar to the one used for intratumoral delivery in this paper (i.e. upto 20 mg flat dose) resulted in considerable lower levels in plasma than those previously reported for a sub-effective lowest tested systemic dose (0.1 mg/kg, Ribas et al. J Clin Oncol 23:8968-77, 2005). Please adjust and discuss.

Some statements in de discussion are rather strong and not entirely justified by the data. For instance, line 12-19 on page 17 (concerning pre-exisitng HLA-restricted anti-tumor immunity) is based on mRNA expression and correlation data, and can only really be claimed when additional protein analyses (such as IHC) and analysis of antigen-specific T cell responses are performed. Please tone down this statement. The statement (rather prominently featured) that fresh samples are superior for immune monitoring is not backed by comparative data sets and distracts from the main message of the paper. The lines 29-31 of page 19 describe a comparison, but it is unclear whether this is a comparison of intratumoral injection to the i.v. control arm, or to historical controls (presumably the latter as the first was NOT a primary endpoint and the trial was not powered for direct comparisons). The very last concluding statement should contain “in the advanced/metastatic setting” as it is not known whether this holds true for earlier stages of disease.

P.17, lines 29-30: was this really unexpected? As the authors themselves point out, activated Tregs (which intratumoral Tregs are) -and their cross-talk with M2 macrophages- may be directly targeted by anti-CTLA4.

We worry that the important message of this paper is lost in the overwhelming amount of data and text, and would advice the authors to make the manuscript more concise and focused. The Results section and Discussion are very long (and the latter too speculative in places).

Minor comments:

Page 6: “percentage patients that dropped out of the study for various reasons”; percentages are calculated out of all patients for the intratumoral group, but out of all patients in the IV group for the IV group. Please express both groups in the same manner.

Authors show lower plasma levels of nivolumab in the IV group, was there a relationship between these plasma levels and clinical response? The authors hypothesize that the unexpected decrease in nivolumab plasma levels in the IV group is due to inflammation-induced clearance, however, we would like to propose that alternatively the PD-1 receptor expression might be higher (up-regulated) in IV-treated patients, and therefore more nivolumab might have been bound to cellular targets.

Why did the authors choose a dose calculated per weight for the local intratumoral administration, instead of a flat dose? Can they explain the rationale for this, and the selection of the dose level?

The presented data on gating strategies, especially concerning (activated) Tregs appear to have some inconsistencies and are very confusing; in every figure a different strategy appears to be used. Can there be a clear definition, more detailed description and rationale of the chosen strategies for tumor versus blood. E.g. it appears that the frequency in whole blood was based solely on CD4/CD127/CD25 (and not FoxP3/CTLA4?)

There is a lack of description on the practical issues concerning the local injections. What was the injected volume? Was there a minimum tumor size as a consequence of the injected volume? How was the injection experienced by the patient (in terms of discomfort), was local anesthesia provided? Did the volume stay in the lesion, or did it flow back out due to internal tumoral pressure?

The authors show data from secretome analyses of tumor pieces. We are concerned that these analyses are presented as quantitative, rather than qualitative, even though the tumor pieces were not necessarily similar in size or cell content. Please include comment/discuss.

Minor language issues: in figure 1d there is the French word stade, instead of English stage, and in the abstract (line 11) “hypothesis” should be “hypotheses”.

Referee #3

(Remarks to the Author)
I co-reviewed this manuscript with one of the reviewers who provided the listed reports.

Referee #4

(Remarks to the Author)
Overall, the manuscript “Safety and Efficacy of Intratumoral Anti-CTLA4 with Intravenous Anti-PD1” by Dr. Tselikas and colleagues represents an impressive and significant contribution to the field of cancer immunotherapy, offering unprecedented and important insights into the comparative safety and mechanisms of efficacy of intratumoral versus intravenous ipilimumab in combination with intravenous nivolumab for the treatment of advanced melanoma.

Summary of the key results:
The single most striking achievement is the successful attainment of the primary endpoint: intratumoral ipilimumab resulted in a dramatically lower incidence of grade 3/4 immune-related adverse events (irAEs) compared to the conventional intravenous route—22.6% versus 57.1% at 6 months, with a toxicity profile more comparable to that of anti-PD1 monotherapy than the doublet.

Originality and significance:
While smaller, single-arm studies have hinted at this previously, this is both the largest –and only randomized– trial, now demonstrating this improved safety and will be the seminal study moving forwards. This achievement establishes this approach as a safer alternative, especially relevant for patient populations with increased risk of severe autoimmune complications and those otherwise unable or unwilling to tolerate high-grade toxicities associated with standard intravenous checkpoint blockade. This study will also be the cited rationale for future (and some ongoing) trials of intratumoral ipilimumab use in both metastatic and (likely neo-adjuvant treatment of) early-stage, high-risk melanoma, and possibly other tumors.
Additionally, the significance of the study is that, in real-world practice, there are patients and physicians who already believe in the benefit of intratumoral therapy, despite the lack of (prior) randomized trials and provide them off-label (e.g. Intratumoral Immunotherapy - Williams Cancer Institute). Seminal trials, such as presented herein, are an opportunity to provide high-level evidence answers to questions patients are already asking.

Data & methodology:
The quality and presentation of data are incredibly thorough, as is appropriate for a high-impact study of this caliber.
The randomized Phase 1b design included well-balanced patient arms reasonably minimizing selection biases.
The authors correctly clarify that the trial is not statistically powered to compare systemic efficacy between the intratumoral and intravenous administration routes, but present efficacy outcomes on both injected and non-injected lesions showing encouraging response rates, these data are interpreted with appropriate caution, avoiding unsupported direct claims of systemic comparability. Their conservative statistical approach is credible and respects the limitations inherent to early phase studies.

Conclusions:
The manuscript’s conclusions are valid and well-justified from the data presented.
Practically, the manuscript provides compelling evidence that intratumoral ipilimumab can be safely administered, even to deep-seated lesions, without local or procedural grade 3 adverse events. This is a critical point which may warrant further emphasis, as academics and industry leaders unfamiliar with the field might perceive such potential adverse events as significant obstacles to the approach.

Suggested improvements:
Overall, no additional experimentation should be required for publication, several specific analyses/comparisons that would help the manuscript are detailed below.

References:
The References are thorough and appropriate with several concerns expressed below.
Clarity and context: The manuscript overall, including abstract, introduction, and conclusions are very well done, with the few concerns expressed below:
The authors skillfully contextualize their results within the broader landscape of ipi/nivo for advanced-stage melanoma.
In all, the manuscript is highly impactful and impressively executed; the intratumoral approach shown herein has immediate clinical relevance for vulnerable patient groups, and the authors outline a clear vision for future trials where this technique may minimize barriers and expand access to checkpoint blockade therapy. This work is a commendable step forward in

personalizing immunotherapy and optimizing risk-benefit profiles for diverse cancer populations.

MAJOR CONCERNS
Regarding data inclusion, the manuscript is fairly rigorous, however, there are significant exclusions which would strengthen the data:
1) The authors currently provide thorough immune correlative data comparisons between Durable Clinical Benefit (DCB) pts and non-DCB, which is illuminating as to mechanisms of efficacy (though a bit confusing since we sometimes use the DCB acronym to mean 'double checkpoint blockade'), however the authors should also include (in either primary or supplementary Figures) immune correlative data comparisons between the intratumoral and intravenous cohorts, regardless of whether statistically significant differences were observed (noting that the study was not powered for these differences).

2) Given prior data demonstrating the predictive power of genomic instability, e.g. somatic copy-number alterations being associated with protumoral inflammation as well as resistance to PD-1 blockade, [Danlos et al. Cancer Discov. 2023] it would be of high interest to know whether these associations did or did not occur in the current study. This is of particular importance because the Danlos study demonstrated that protumoral inflammation either correlated with or caused Treg infiltration, which the current study (with i.t. or i.v. CTLA4 blockade) has now flipped to be a predictor of favorable clinical outcome.

3) The authors should be more precise and emphatic as to how this trial addresses another often-raised concerns regarding intratumoral therapy:
For example, regarding the concern whether the approach is sufficiently practical and such trials can be feasibly accrued/completed, the authors should clarify the total enrollment time per site (it appears that 2 sites were only opened for the latter part of the trial), and potentially put this in the context of a relevant comparator e.g. the MASTERKEY-265 trial which used intratumoral administration of oncolytic virus, accruing 700 patients at only 18 centers in 25 months.
Another example would be clearer data regarding the concern that the intratumoral approach may result in procedural (injection site) adverse events. The authors do indicate that there were no grade 3/4 procedural adverse events, however this data should also be provided for grade 1/2 events, presumably as an additional row in Extended Data Table 2.

4) For the readership of Nature, the authors should present more clinical context in Introduction or Discussion for relevant comparators of nivo + i.t. ipi e.g. how did their results compare to the seminal trial of nivo + anti-LAG-3 [Tawbi HA et al, NEJM 2022] per safety and efficacy metrics (i.e. PFS).

5) Again, for clinical context, the significance of studying nivo + i.t. ipi is only as great as the unmet need with the standard therapy (nivo + i.v. ipi). The manuscript would be strengthened by providing some data regarding what proportions of patients actually receive (nivo + i.v. ipi) in real world datasets e.g. those provided by [C T van Zeijl, M et al., J Immunother. 2023] or [Klein-Brill A et al., BJC Rep. 2024] or other sources.

MINOR CONCERNS
-The term "Biologic abnormalities" in Extended Data Table 2 is unclear and, particularly as there are 11 patients with Grade 3/4 "Biologic abnormalities" (between the two arms) this should explained in some granular detail.
-Interleukin-2 is misspelled "Interleukine-2"
-The authors do not seem to cite the most relevant study for clinical context, the 10-year update of Checkmate 067 – Wolchok J et al., N Engl J Med. 2025 (instead citing the Wolchok JCO 2022 update), this should be fixed. This is critical, as the latest (10-year) follow up indicates that there is still no survival benefit to intravenous nivo+ipi as compared to nivo alone. This is particularly important for the Nature readership (as opposed to Lancet or NEJM) that may not be aware that the 'gold standard' (intravenous) therapy is associated with high toxicity but not prolonged survival.
-The manuscript notes that a prior study of intratumoral ipilimumab [Ray et al Oncotarget . 2016] observed significant injection site reactions / ulcerations in 5 of 12 patients, though none were observed in this study. Given that large discrepancy, it would be helpful if the authors could present some hypothesis (or data) attempting to explain the difference.

-It is of some concern that the Fc Receptor data (Fig 6H-I) demonstrating improved clinical outcomes for patients whose tumors contain higher a priori levels of FCGR1A, FCGR3A, FCGR3B is obtained from transcriptomics, it would be strengthened by corroborative protein-level data if available.

Version 1:

Reviewer comments:

Referee #1

(Remarks to the Author)
After reviewing the authors' detailed rebuttal and the revised manuscript, I confirm that all comments raised during peer review have been fully addressed. The authors provided appropriate additional analyses, clarified key mechanistic claims, and improved the manuscript's clarity and structure. In light of these revisions, I consider the manuscript suitable for publication and have no further concerns.

Referee #2

(Remarks to the Author)
The authors have addressed all our concerns adequately. We do still feel the manuscript is rather long, but certainly acceptable for publication.

Referee #3

(Remarks to the Author)
I co-reviewed this manuscript with one of the reviewers who provided the listed reports.

Referee #4

(Remarks to the Author)
The Critiques included in my prior Review were well and quite thoroughly addressed in the Response and with appropriate Revisions to the manuscript. I would recommend publication without further edits.

Open Access This Peer Review File is licensed under a Creative Commons Attribution 4.0 International License, which permits use, sharing, adaptation, distribution and reproduction in any medium or format, as long as you give appropriate credit to the original author(s) and the source, provide a link to the Creative Commons license, and indicate if changes were made.
In cases where reviewers are anonymous, credit should be given to 'Anonymous Referee' and the source.
The images or other third party material in this Peer Review File are included in the article's Creative Commons license, unless indicated otherwise in a credit line to the material. If material is not included in the article's Creative Commons license and your intended use is not permitted by statutory regulation or exceeds the permitted use, you will need to obtain permission directly from the copyright holder.
To view a copy of this license, visit https://creativecommons.org/licenses/by/4.0/

Safety and Efficacy of Intratumoral Anti-CTLA4 with Intravenous Anti-PD1

- Nature manuscript 2025-08-21480 -

Point-by-point Response to our Referees

First, we would like to thank our referees for the time they have dedicated to review our manuscript.

We believe that their comments and requests have further improved the quality of our manuscript and strengthened its message.

Referee #1 (Remarks to the Author):

This manuscript reports the results of a randomized Phase 1b trial (NCT02857569), evaluating the safety and efficacy of intratumoral (IT) ipilimumab (0.3 mg/kg) combined with intravenous (IV) nivolumab (1 mg/kg), compared to the standard IV ipilimumab (3 mg/kg) plus nivolumab, in treatment-naive patients with advanced melanoma. The study met its primary endpoint, showing reduced grade ≥3 irAEs in the IT arm (22.6%) compared to the IV arm (57.1%). The manuscript also presents an extensive translational program, identifying predictors of durable clinical benefit (DCB), notably the presence of intratumoral activated Tregs and M2 macrophages.

General Comments

The study addresses a critical clinical issue: reducing anti-CTLA4-related toxicities while maintaining efficacy. Intratumoral delivery of ipilimumab is innovative and potentially practice-changing. The study showed a marked reduction in systemic toxicity and extensive translational data set (biopsies, secretome, flow cytometry).

Reply to Referee #1:

We thank **Referee #1** for his expertise and the time spent to review our work. We appreciate his comments characterizing our results as potentially practice-changing.

Referee #1:

Efficacy comparison underpowered but informative. While not powered for efficacy, the IT arm showed a 50% response in non-injected lesions, close to the 62% in the IV arm. It would be good to stratify responses by injection site or lesion type, if possible.

Reply to Referee #1:

We thank Referee #1 for this important question. We performed the analysis only on most frequent lesions (n>10) in order to generate relevant descriptive statistics. We found more CR in skin lesions than in lymph node lesions, but overall the objective response rate was slightly higher in lymph nodes (see below).

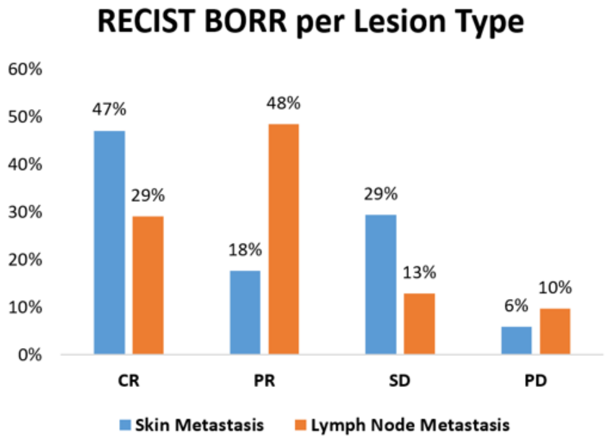

BORR (CR+PR) per lesion type

- Skin Metastasis = 64.7% (11/17)
- Lymph Nodes Metastasis = 77.4% (24/31)

We have now added the BORR per lesion type to the **Extended Data Figure 1a**.

Referee #1:

Unexpectedly, Tregs and M2 macrophages predicted response (DCB). This highlights the complexity of tumor immune ecosystems. The CD25+CD39+CD4+ Treg characterization via flow cytometry is robust; please, discuss feasibility of using this as a clinical biomarker.

Reply to Referee #1:

We agree with Referee #1 that flow cytometry is a technique that can be used to assess biomarkers in clinical practice. It is already used in academic hospitals for diagnostic purposes in hematological malignancies (e.g phenotype of lymphoma and leukemia) but also to monitore minimal residual disease and stratify patient care (e.g for the therapeutic strategy of lymphoblastic leukemia or in the context of allogeneic bone marrow transplantation). Here the level of activated Tregs in baseline fresh tumor biopsies seems highly predictive of potential benefit of anti-PD1 + anti-CTLA4 immunotherapy.

We believe that the titration of tumor biopsy secretome is also something that can be standardized and used in routine practice because titration of cytokines is technically available and increasingly used in routine practice in academic hospitals (e.g IL6 titration to monitore cytokine release syndromes in the context of CAR-T cells or bispecific T-cell engagers therapies). Here we show that the quantities of Granzyme A and Granzyme B is also highly predictive of potential benefit of anti-PD1 + anti-CTLA4 immunotherapy.

Those biomarkers could indeed be used to stratify patient care in melanoma. Their predictive value need to be assessed in other cancer histologies.

One paragraph in our discussion already addresses those points:

"the measure of CD4+CD25+CD39+ T-cells by flow cytometry on fresh tumor biopsies or the titration of Granzyme A or B in the secretome of these same fresh tumor biopsies represent simple

and rapid techniques to provide robust predictive biomarkers and forecast the therapeutic benefits to anti-PD1 + anti-CTLA4 immunotherapy. The great sensitivity and specificity of biomarkers identified on fresh material assessment is well illustrated in our results by the small overlap at baseline in the ranges of values between patients with subsequent DCB and No DCB for Granzyme B titration in the secretome compared to Granzyme B quantified by IHC." [Page 20; Line 29]

In order to emphasize on the feasibility of such approaches underlined by Referee #1, we have added the following sentence to our Discussion:

"Because flow cytometry (e.g diagnosis and monitoring of minimal residual disease in lymphoblastic leukemias) and titration of cytokines (e.g IL-6 titration to monitor cytokine release syndromes induced by CAR-T cells) are already used in routine practice in academic hospitals for the treatment of hematological malignancies, they could also be used tomorrow for the treatment of solid malignancies." [Page 20; Line 33]

Referee #1:

Pharmacokinetics show lower ipilimumab levels in the IT arm. Nivolumab levels were paradoxically lower in the IV ipilimumab group. This finding adds mechanistic insight into immune activation versus toxicity.

Reply to Referee #1:

Please see reply to **Referee #2** on that point.

Referee #1:

Minor Comments

Abstract: Consider clarifying the sentence on cross-reactivity (line 9).

Reply to Referee #1:

The sentence has been clarified accordingly:

"stronger activation of tumor infiltrating T-cells could increase systemic toxicity in case of cross-reactivity of their T-cell receptor with healthy tissue antigens." [Page 3; Line 9]

Referee #1:

Figures: Consider summarizing safety/efficacy trade-offs in a new schematic figure.

Reply to Referee #1:

Per Referee #1's request we have generated a new figure which could serve as a graphical summary of our results. This figure could support future oral presentations.

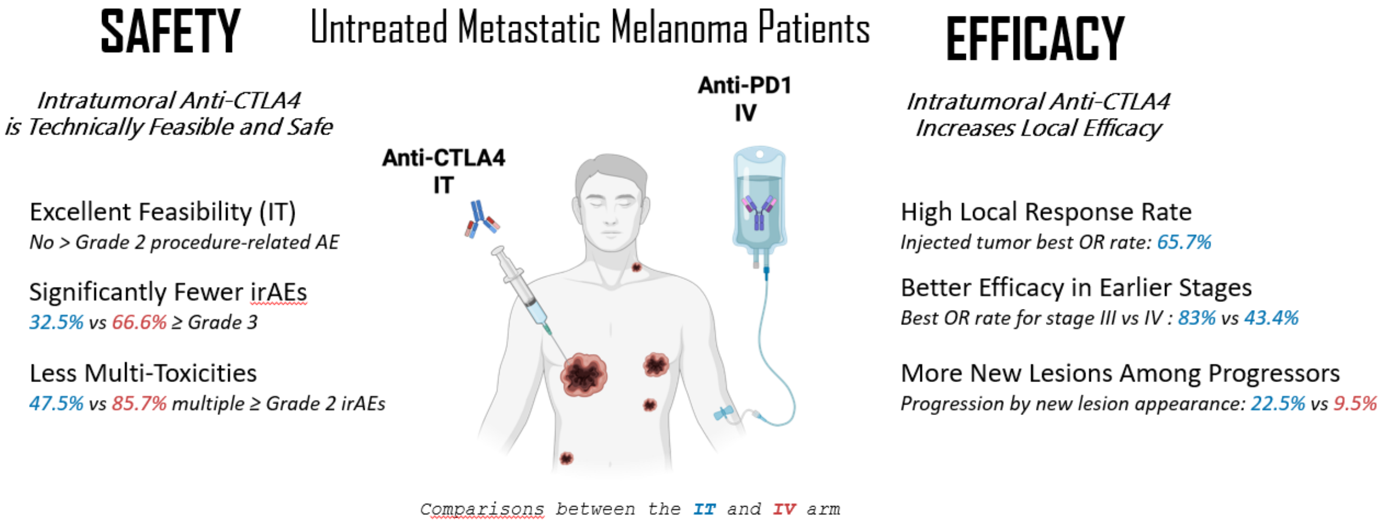

It has been added as Extended Data Figure 8.

Referee #1:

Extended Data: Move key Treg depletion figure (Ext Data Fig 6) to main figures.

Reply to Referee #1:

Extended Data Figure 6f has been moved to Figure 6e (right) as requested.
The corresponding figure legends have been modified accordingly.

Referee #1:

Typo in abstract: "those hypothesis" → "these hypotheses".

Reply to Referee #1:

We thank Referee #1 for noting this mistake. The plural has been corrected.

Referee #2 (Remarks to the Author):

The manuscript "Safety and Efficacy of Intratumoral Anti-CTLA4 with Intravenous Anti-PD1" by Tselikas et al. is a very impressive body of work describing a timely study in the exciting field of intratumoral immunotherapy. It shows the safety and clinical efficacy of i.t. anti-CTLA4 and i.v. anti-PD-1 in advanced melanoma to be equivalent to i.v. Although a non-significant advantage in terms of survival was found in the i,v, arm.

We are very excited about this manuscript. The described study is highly original and innovative and we strongly feel that it should be published to the benefit of the cancer immunotherapy field. Lower irAE rates combined with optimal efficacy would facilitate wider application of this powerful dual immunotherapy, particular in earlier disease stages and the neoadjuvant setting.

Overall, the approach is valid and the quality of the presented data is high. The used statistics are appropriate. Overall the conclusions are robust and justified. Nevertheless, some statements are an over-interpretation; we have some comments and feedback to further improve the quality of the work and help showcase the very important results of this study more effectively, see below.

Reply to Referee #2:

We thank Reviewer#2 for his thorough assessment of our manuscript and his favorable review of our work.

Referee #2:

Major comments:

In the immunomonitoring section, we think more emphasis should be placed on the comparison between intratumoral and intravenous administration, as this was the aim of the trial and paper. The fact that no, or very little difference can be found in immune response between the two different strategies is of profound interest, as this proves that intratumoral delivery of anti-CTLA4 equally and similarly effects immune activation as intravenous administration.

Reply to Referee #2:

We agree with Referee #2 that the comparison of tumors between IT and IV treated patients between baseline and on-treatment is an important point to address. We have added the following paragraph at the end of the Results section and have pooled those comparisons in the additional Extended Data Figure 7 (see below):

"Of note, although we observed higher markers of immune activation in the periphery upon high IV ipilimumab, there was no specific difference in tumors whether the anti-CTLA4 was injected IT or IV for total intratumoral T cells (Extended Data Fig. 7a). An increase of CD8 T cells in the

tumor microenvironment upon treatment was not obvious by RNAseq (Extended Data Fig. 7b left), but was more significant per IHC staining (Extended Data Fig. 7b middle) and by flow cytometry for the subset of activated CD8+ T cells (Extended Data Fig. 7b right). No differences were seen overall whether ipilimumab was injected IT or IV for intratumoral Tregs (Extended Data Fig. 7c), and CD68 expression (Extended Data Fig. 7d). But the expression of CD163 was increasing upon treatment in both the IT and IV treated patients (Extended Data Fig. 7e), and there was a trend for upregulation of FcgR upon treatment in both arms for FCGR1A, FCGR3A and FCGR3B (Extended Data Fig. 7f left, middle and right panels respectively)." [Page 14; Line 27]

Extended Data Figure 7

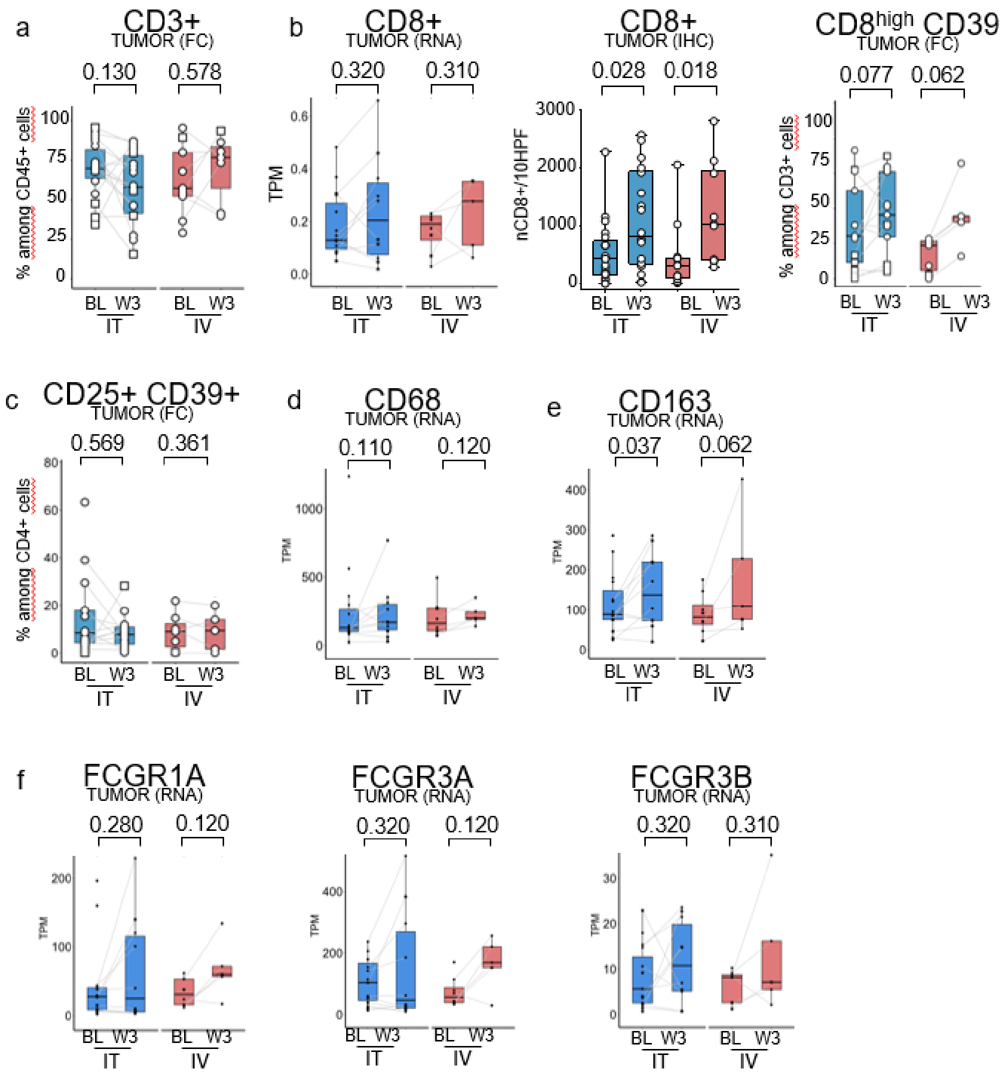

Referee #2:

We miss the likely role of the tumor-draining lymph nodes in the efficacy of intratumoral injection in the discussion of the manuscript. The authors discuss that they have two hypotheses for the clinical responses observed in the non-injected lesions; one is optimal exposure of the TME (but what about TDLN?) The other that the intratumorally injected dose leaks into the periphery, and they use a reference to back this claim. However, in that referenced publication, a similar dose was injected systemically and intratumorally, whereas in this study a tenfold lower dose was used intratumorally. As such this hypothesis is not backed by the listed reference. In another study of locally injected tremelimumab (van Pul et al. Sci Immunol 7: eabn8097 2022) dose levels similar to the one used for intratumoral delivery in this paper (i.e. upto 20 mg flat dose) resulted in considerable lower levels in plasma than those previously reported for a sub-effective lowest tested systemic dose (0.1 mg/kg, Ribas et al. J Clin Oncol 23:8968-77, 2005). Please adjust and discuss.

Reply to Referee #2:

We agree with referee #2 that the tumor draining lymph nodes could play a role in the systemic immune changes observed in our patients. We have added this possibility in the discussion:

"Third, the role of the tumor draining lymph nodes, could be critical in the potential systemic effects of local anti-CTLA4 therapy. In accordance with the hypothesis of a potential role of the tumor draining lymph nodes, Van Pul et al have shown, in the context of adjuvant therapy of resected localized melanoma, that intradermal low doses (2-20mg) of tremelimumab (IgG2 anti-CTLA4) resulted in significant systemic immune changes on circulating activated Tregs and subsets of myeloid cells[42]." [Page 17; Line 19]

We have added the corresponding citation as reference :

van Pul, K. M. et al. Local delivery of low-dose anti–CTLA-4 to the melanoma lymphatic basin leads to systemic Treg reduction and effector T cell activation. Sci. Immunol. ***7****, eabn8097 (2025).*

The potential role of the tumor draining lymph node has also been added further in the discussion to explain the systemic pharmacodynamic changes seen upon intratumoral anti-CTLA4.

"…the systemic pharmacodynamic effects of ipilimumab could be the consequence of exposure to high dose of anti-CTLA4 (either via direct IT injection or systemic high dose) of the tumor microenvironment and/or its draining lymph nodes." [Page 18; Line 5]

Referee #2:

Some statements in the discussion are rather strong and not entirely justified by the data. For instance, line 12-19 on page 17 (concerning pre-exisitng HLA-restricted anti-tumor immunity) is based on mRNA expression and correlation data, and can only really be claimed when additional protein analyses (such as IHC) and analysis of antigen-specific T cell responses are performed. Please tone down this statement.

Reply to Referee #2:

We believe that our statement on the predictive role of pre-exisitng HLA-restricted anti-tumor immunity associated with clinical benefit upon anti-PD1 + anti-CTLA4 is supported by the significant differences found at baseline for HLA genes and proteins (Figure 5d&e), and the significant differences found for CD8+ PD1+ T-cells (Figure 5c) and B-cells (Figure 5g) both at the RNA and protein levels. However, we agree with Referee #2 that future work could be done to further demonstrate how pre-existing adaptive anti-tumor immune responses are at play at baseline and how they are modified upon checkpoint targeted therapy notably with studies on antigen specific responses, TCR clonality and phenotype of effector cells.

We have dampen our statement to take into consideration the relevant remark from Referee #2 that more work should be done in the future on that question of tumor specific adaptive anti-tumor immune responses.

The corresponding sentence now reads:

"*First, we found that higher baseline MHC-I and MHC-II expressions and higher abundance of adaptive immune effector cells was associated with favorable outcomes upon anti-PD1 and anti-CTLA4 therapy*." *[Page 18; Line 18]*

Referee #2:

The statement (rather prominently featured) that fresh samples are superior for immune monitoring is not backed by comparative data sets and distracts from the main message of the paper. The lines 29-31 of page 19 describe a comparison, but it is unclear whether this is a comparison of intratumoral injection to the i.v. control arm, or to historical controls (presumably the latter as the first was NOT a primary endpoint and the trial was not powered for direct comparisons). The very last concluding statement should contain "in the advanced/metastatic setting" as it is not known whether this holds true for earlier stages of disease.

Reply to Referee #2:

We agree with Referee #2 that the value of biomarkers generated with fresh tissue was not a primary endpoint of our trial. However we believe that the prospective collection of such data and its strong predictive value is an important demonstration of feasibility and relevance of using fresh

tissue for future biomarkers in IO. In our text (initially lines 29-31 of page 19) we were referring to the great difference between baseline values of granzymes by secretome titration between DCB and No DCB groups (Figure 5f) vs the inability of baseline granzymes as measured by IHC to provide such predictive value (Extended Data Fig.5f).

In order to take into consideration both Referee #1 and 2 requests while not expanding the length of that section of the discussion, we have removed the following paragraph:

"~~The great sensitivity and specificity of biomarkers identified on fresh material assessment is well illustrated in our results by the small overlap at baseline in the ranges of values between patients with subsequent DCB and No DCB for Granzyme B titration in the secretome compared to Granzyme B quantified by IHC~~." [Removed from initial Page 20; Line 11]

Also with have put conditional in our statements as we agree with Referee #2 that our discoveries need further prospective validations:

"*Another important finding of our study is the **potential** value of fresh tumor sample analysis to identify predictive biomarkers …" [Now Page 20; Line 16]*

Also we have added this statement at the end of that section of the discussion:

"However the predictive value of those novel biomarkers will need to be confirmed in validation cohorts." [Now Page 21; Line 3]

Referee #2:

P.17, lines 29-30: was this really unexpected? As the authors themselves point out, activated Tregs (which intratumoral Tregs are) -and their cross-talk with M2 macrophages- may be directly targeted by anti-CTLA4.

Reply to Referee #2:

The finding that higher Tregs and M2 macrophages were found selectively in DCB patients was indeed unexpected by us and we believe that the vast majority of our community would indeed expect these cells to be higher in the No DCB patient subgroup.

Referee #2:

We worry that the important message of this paper is lost in the overwhelming amount of data and text, and would advice the authors to make the manuscript more concise and focused. The Results section and Discussion are very long (and the latter too speculative in places).

Reply to Referee #2:

As emphasized by our referees, our work contains numerous significant results and our ancillary analyses have been comprehensive. We have tried to reduce the length of our manuscript while addressing our reviewers's requests. We are happy to further work with the editorial team to reduce the number of words if deemed necessary.

Referee #2:

Minor comments:

Page 6: "percentage patients that dropped out of the study for various reasons"; percentages are calculated out of all patients for the intratumoral group, but out of all patients in the IV group for the IV group. Please express both groups in the same manner.

Reply to Referee #2:

Denominators of the number of patients from the IT arm have now been added [page 6; line 16].

Referee #2:

Authors show lower plasma levels of nivolumab in the IV group, was there a relationship between these plasma levels and clinical response?

Reply to Referee #2:

We agree with Referee #2 that the question of nivolumab exposure and clinical outcome is relevant following our observation that systemic high dose ipilimumab is associated to significant lower circulating concentrations of nivolumab. However we believe that we don't have enough patients at risk in our IV arm to answer this question.

Referee #2:

The authors hypothesize that the unexpected decrease in nivolumab plasma levels in the IV group is due to inflammation-induced clearance, however, we would like to propose that alternatively the PD-1 receptor expression might be higher (up-regulated) in IV-treated patients, and therefore more nivolumab might have been bound to cellular targets.

Reply to Referee #2:

We and others have shown that inflammation can increase the clearance anti-CTLA4 and anti-PD1, and has a negative impact on overall survival :

- Wang, E. et al. Population Pharmacokinetic and Pharmacodynamic Analysis of Tremelimumab in Patients With Metastatic Melanoma. J. Clin. Pharmacol. 54, 1108–1116 (2014).
- Wang, R. et al. Development of a prognostic composite cytokine signature based on the correlation with nivolumab clearance: translational PK/PD analysis in patients with renal cell carcinoma. J. Immunother. cancer 7, 348 (2019).
- de Vries, F. et al. Early pembrolizumab clearance as prognostic biomarker for non-response in patients with advanced non-small cell lung cancer. Int. J. cancer 157, 2569–2576 (2025).
- Maritaz, C. et al. Nivolumab plasma concentration and clearance associated with overall survival in patients with renal cell carcinoma. J. Immunother. cancer 13, (2025).

Referee #2 proposes that the decreased levels of circulating nivolumab could be a consequence of an upregulation of PD1 protein membrane expression which thereby could be responsible of a sink effect for the circulating levels of nivolumab.

In order to assess the question of PD1 saturation in the blood, we were using the anti-PD1 staining antibody clone EH12-2H7 that is competitive with nivolumab epitope. At the end of the first cycle (W3), we found a significant saturation of PD1 on circulating CD4 and CD8 T-cells:

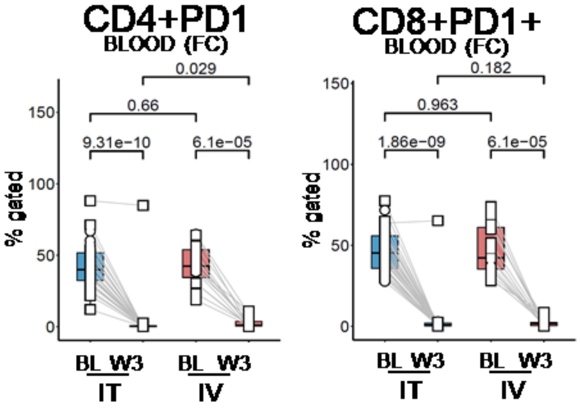

Also, the soluble PD-1 increase (which is acting as a potential decoy receptor) was increased at similar levels in the two arms of the study:

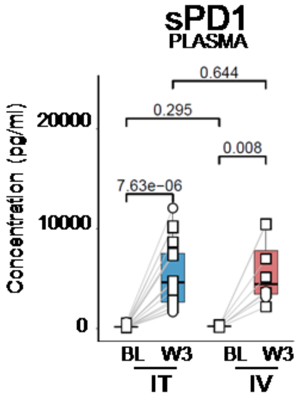

However when comparing the proportions of PD1+ CD4 and CD8 T cells at W3, we found more positive cells at W3 in the IV arm:

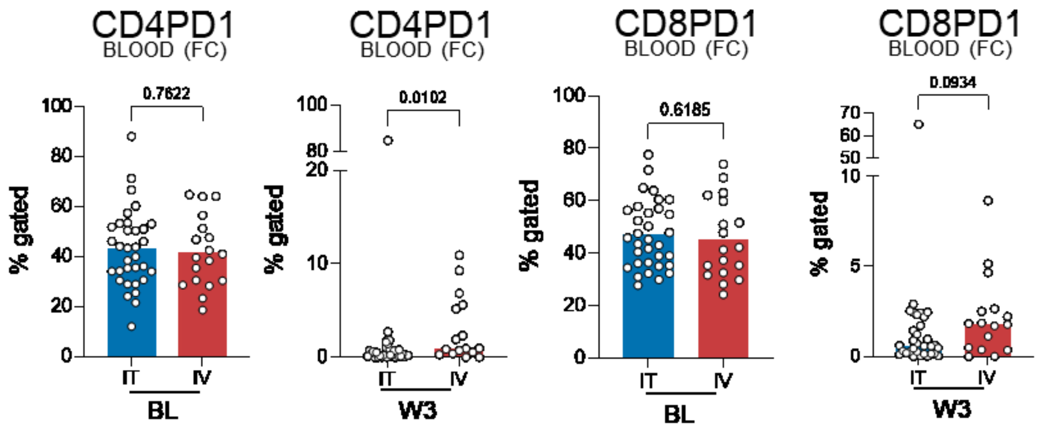

At the MFI level, which reflects the quantity of PD1 proteins expressed at the membrane of cells, we found only a higher MFI for the CD4+ T cells but not for CD8+ T cells in patients treated in the IV arm:

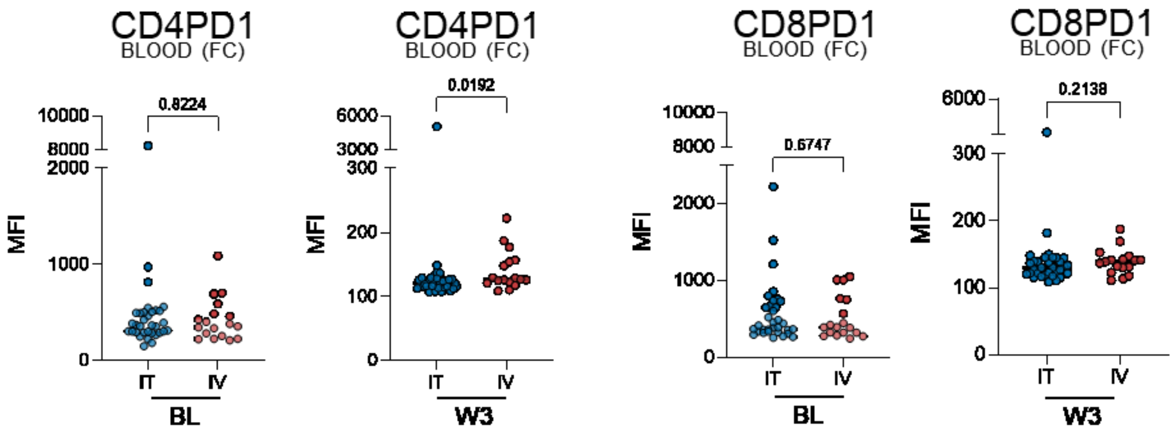

Because we were using a PD1 staining antibody clone competitive with nivolumab, those results suggest that some patients in the IV arm did not reach the same level of PD1 saturation than patients in the IT arm.

However this approach could not answer specifically if the PD1 expression is upregulated upon ipilimumab IV.

In the flow cytometry panels used for the analysis of the tumor biopsies, we were using another PD1 staining clone (MIH4) that is NOT competing with nivolumab epitope therefore assessing the PD1 epression independently from the presence of nivolumab exposure.

Here we did not find significantly more proportions of PD1+ CD4 and CD8 T cells upon IV ipilimumab. We found actually less PD1 on tumor infiltrating T-cells in the IT arm suggesting an

internalization of PD1 upon IT anti-CTLA4, in line with what has been observed by PD1xCTLA4 bispecific antibodies(e.g: *Dovedi SJ, et al. Design and Efficacy of a Monovalent Bispecific PD-1/CTLA4 Antibody That Enhances CTLA4 Blockade on PD-1+ Activated T Cells. Cancer Discov. 2021 May;11(5):1100-1117. doi: 10.1158/2159-8290.CD-20-1445. Epub 2021 Jan 8. PMID: 33419761*.):

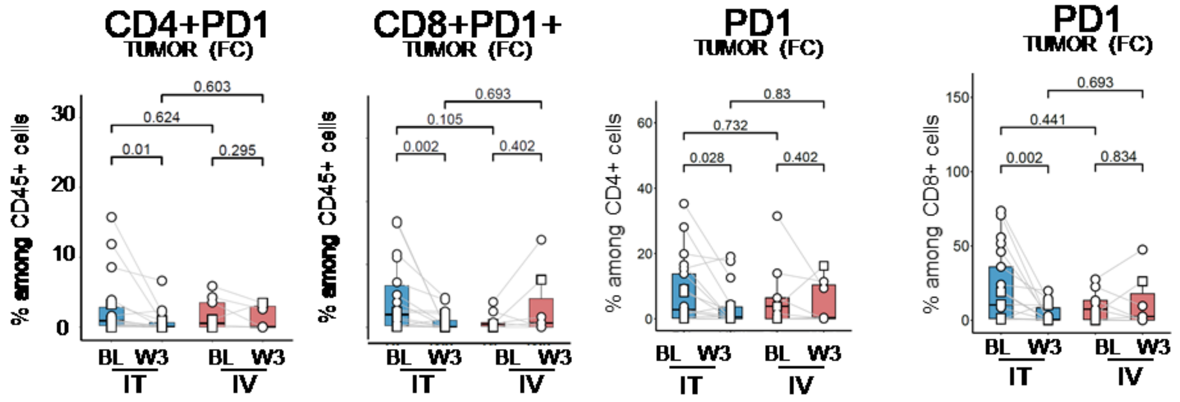

Altogether our results suggest that indeed there is a statistically significant lower nivolumab exposure upon ipilimumab IV with statistically less PD1 saturation in the blood and no increase of PD1+ T cells in the tumor. However, those numerical differences might not be clinically relevant as the efficacy observed in the IV arm of the study remained good (the lower nivolumab exposure could be compensated by the higher ipilimumab exposure).

Although interesting, we have not added this additional dataset to our manuscript because we believe that those observations do not add much on top of what has been already published in the above mentioned references: a higher clearance of checkpoint antibodies can have a detrimental impact on exposure and therefore on efficacy and survival. Our PK data in Figure 2g already illustrates that and suggest that patients receiving ipilimumab IV could benefit from a higher dose of nivolumab.

Referee #2:

Why did the authors choose a dose calculated per weight for the local intratumoral administration, instead of a flat dose? Can they explain the rationale for this, and the selection of the dose level?

Reply to Referee #2:

The rationale was based on the pre-clinical dataset published in Marabelle et al. JCI 2013 (see reference below; ref #44 of the main manuscript) where 1/10th and 1/100th intratumoral anti-CTLA4 were still generating anti-tumor efficacy. We chose 1/10th to avoid a step of dilution and work with pure ipilimumab IT and in order to work with ipilimumab volumes amenable to IT injections (0.3mg/kg represents ~20mg for a 70kg adult and a volume of 4mL to be injected with the commercial ipilimumab concentration at 5mg/mL).
For subsequent trials we have moved to flat doses (e.g PRIMO trial, NCT04270864 with ipilimumab 20mg flat dose IT).

Ref #44 :
Marabelle, A. et al. Depleting tumor-specific Tregs at a single site eradicates disseminated tumors. J. Clin. Invest. ***123****, 2447–2463 (2013).*

Referee #2:

The presented data on gating strategies, especially concerning (activated) Tregs appear to have some inconsistencies and are very confusing; in every figure a different strategy appears to be used. Can there be a clear definition, more detailed description and rationale of the chosen strategies for tumor versus blood. E.g. it appears that the frequency in whole blood was based solely on CD4/CD127/CD25 (and not FoxP3/CTLA4?)

Reply to Referee #2:

Blood Tregs were indeed identified as viable CD45+CD3+CD4+CD25+CD127- as validated by the following references:

- *Seddiki, N. et al. Expression of interleukin (IL)-2 and IL-7 receptors discriminates between human regulatory and activated T cells . J. Exp. Med. 203, 1693–1700 (2006).*
- *Liu, W. et al. CD127 expression inversely correlates with FoxP3 and suppressive function of human CD4+ T reg cells . J. Exp. Med. 203, 1701–1711 (2006).*
- *Shen, L.-S. et al. CD4(+)CD25(+)CD127(low/-) regulatory T cells express Foxp3 and suppress effector T cell proliferation and contribute to gastric cancers progression. Clin. Immunol. 131, 109–118 (2009).*
- *Yu, N. et al. CD4(+)CD25 (+)CD127 (low/-) T cells: a more specific Treg population in human peripheral blood. Inflammation 35, 1773–1780 (2012).*

Our gating strategy for blood flow cytometry was referenced in the Methods section under the paragraph "*Flow Cytometry Immune Cells Phenotyping and Analysis from blood samples*" together with the list of antibody clones used for staining (Methods Table 2). This gating strategy was published here:

Pitoiset, F. et al. Deep phenotyping of immune cell populations by optimized and standardized flow cytometry analyses. Cytometry A ***93****, 793-802 (2018)*

Intratumoral Tregs were identified as viable CD45+CD3+CD4+CD25+CD39+ as validated by the following references

- *Bredel, D. et al. Immune checkpoints are predominantly co-expressed by clonally expanded CD4+FoxP3+ intratumoral T-cells in primary human cancers. J. Exp. Clin. Cancer Res. 42, 333 (2023).*
- *Mandapathil M., et al. Increased Ectonucleotidase Expression and Activity in Regulatory T Cells of Patients with Head and Neck Cancer. Clin. Cancer Res. 2009;15:6348–6357.*

- *Ahlmanner F., et al. CD39+ Regulatory T Cells Accumulate in Colon Adenocarcinomas and Display Markers of Increased Suppressive Function. Oncotarget. 2018;9:36993–37007.*
- *Kortekaas K.E., et al. CD39 Identifies the CD4+ Tumor-Specific T-Cell Population in Human Cancer. Cancer Immunol. Res. 2020;8:1311–1321.*

Our gating strategy for tumor samples was illustrated in The Methods section under the paragraph "*Flow Cytometry Immune Cells Phenotyping and Analysis from tumor biopsies*" which contains FACS dot plots to illustrate the gating strategy. Also, the Extended Data Fig 6a further provides an illustration of the FOXP3 and CTLA4 protein expression levels in melanoma according to CD25 and CD39 co-expression.

For clarity, we have simplified the panel illustrations and legends of Methods Figure 2 but let the detailed list of antibody clones used for staining.

Referee #2:

There is a lack of description on the practical issues concerning the local injections. What was the injected volume?

Reply to Referee #2:

We specified in the introduction that the commercial ipilimumab at 5mg/mL was used for direct injection. We have added this information to the "Treatment and follow-up scheduled / Experimental IT Arm" section of the Methods:

"Pure ipilimumab from commercial vials at 5mg/mL were used for IT injections. For instance, a 75kg patients would receive 75x0.3=22.5mg, i.e 4.5mL of pure ipilimumab into the chosen tumor lesions.." [page 36; line 20]

Referee #2:

Was there a minimum tumor size as a consequence of the injected volume?

Reply to Referee #2:

We have added this information to the "Treatment and follow-up scheduled / Experimental IT Arm" section of the Methods:

"Minimal size of injected lesions was 1cm in diameter per protocol. The volume of ipilimumab per patient could be divided in order to inject several lesions but with the objective of adjusting the injected volume to the volume of the treated lesion as much as possible.." [page 36; line 28]

Referee #2:

How was the injection experienced by the patient (in terms of discomfort), was local anesthesia provided? Did the volume stay in the lesion, or did it flow back out due to internal tumoral pressure?

Reply to Referee #2:

Further details have been added to that section of the Methods in order to provide more practical details about how the injection were performed and experienced by patients:

"All injections were performed under image guidance by interventional radiologists using 22 gauge (22G) Chiba needles with a single endhole. The injected volume ranged between 3 and 6.5ml (5mg/ml), and a different part of the tumor was injected at each cycle in a clockwise manner.
A very low injection rate was used (1ml/min) to avoid reflux and optimize distribution within the tumor. Local anesthesia with lidocaine (2%) was used for all excepted for some superficial skin lesions." [page 36; line 22]

Referee #2:

The authors show data from secretome analyses of tumor pieces. We are concerned that these analyses are presented as quantitative, rather than qualitative, even though the tumor pieces were not necessarily similar in size or cell content. Please include comment/discuss.

Tumor biopsies were standardized by the size of the core collected (18G) and the volume of dilution (1mL of saline) in which they were incubated for 24h. Titrations were quantitative using ultra sensitive multiplex assays from Meso Scale Discovery. Regarding variability, the 18G biopsy turn out to be a stable quantity of tumor material (the weight is always around 0.02g, and is stable across weighed biopsies). We found that there is no need to standardize for biopsy weight or secreted protein quantity. Indeed, small variations in tumor material in the biopsy core have negligible consequences given the volume of saline dilution used (1mL) and the abundance of factors discriminating between DCB and No DCB patients.

The following paragraph has been added to the "Plasmatic and Secretome Cytokines Measurement" section of the Methods in order to provide those important technical details:

"Low platelet plasma was generated on blood tubes collected in EDTA upon double high speed centrifugation at 4°C. Secretome experiments were standardized following the same procedure: one single 18G fresh core needle biopsy was put into 1ml saline an incubated at 37°C for 24h prior collection of the supernatants." [page 43; line 2]

Referee #2:

Minor language issues: in figure 1d there is the French word stade, instead of English stage, and in the abstract (line 11) "hypothesis" should be "hypotheses".

Reply to Referee #2:

We thank Referee #2 for his vigilance; "stage" in Figure 1d and "hypotheses" in the abstract have been corrected accordingly.

Referee #3 (Remarks to the Author):

I co-reviewed this manuscript with one of the reviewers who provided the listed reports.

Reply to Referee #3:

We would like to thank Reviewer #3 for his time and joint effort with another reviewer to review our manuscript.

Referee #4 (Remarks to the Author):

Overall, the manuscript "Safety and Efficacy of Intratumoral Anti-CTLA4 with Intravenous Anti-PD1" by Dr. Tselikas and colleagues represents an impressive and significant contribution to the field of cancer immunotherapy, offering unprecedented and important insights into the comparative safety and mechanisms of efficacy of intratumoral versus intravenous ipilimumab in combination with intravenous nivolumab for the treatment of advanced melanoma.

Summary of the key results:

The single most striking achievement is the successful attainment of the primary endpoint: intratumoral ipilimumab resulted in a dramatically lower incidence of grade 3/4 immune-related adverse events (irAEs) compared to the conventional intravenous route—22.6% versus 57.1% at 6 months, with a toxicity profile more comparable to that of anti-PD1 monotherapy than the doublet.

Originality and significance:

While smaller, single-arm studies have hinted at this previously, this is both the largest –and only randomized– trial, now demonstrating this improved safety and will be the seminal study moving forwards. This achievement establishes this approach as a safer alternative, especially relevant for patient populations with increased risk of severe autoimmune complications and those otherwise unable or unwilling to tolerate high-grade toxicities associated with standard intravenous checkpoint blockade. This study will also be the cited rationale for future (and some ongoing) trials of intratumoral ipilimumab use in both metastatic and (likely neo-adjuvant treatment of) early-stage, high-risk melanoma, and possibly other tumors.

Additionally, the significance of the study is that, in real-world practice, there are patients and physicians who already believe in the benefit of intratumoral therapy, despite the lack of (prior) randomized trials and provide them off-label (e.g. Intratumoral Immunotherapy - Williams Cancer Institute). Seminal trials, such as presented herein, are an opportunity to provide high-level evidence answers to questions patients are already asking.

Data & methodology:
The quality and presentation of data are incredibly thorough, as is appropriate for a high-impact study of this caliber.
The randomized Phase 1b design included well-balanced patient arms reasonably minimizing selection biases.
The authors correctly clarify that the trial is not statistically powered to compare systemic efficacy between the intratumoral and intravenous administration routes, but present efficacy outcomes on both injected and non-injected lesions showing encouraging response rates, these data are interpreted with appropriate caution, avoiding unsupported direct claims of systemic comparability. Their conservative statistical approach is credible and respects the limitations inherent to early phase studies.

Conclusions:
The manuscript's conclusions are valid and well-justified from the data presented.
Practically, the manuscript provides compelling evidence that intratumoral ipilimumab can be safely administered, even to deep-seated lesions, without local or procedural grade 3 adverse events. This is a critical point which may warrant further emphasis, as academics and industry leaders unfamiliar with the field might perceive such potential adverse events as significant obstacles to the approach.

Suggested improvements:
Overall, no additional experimentation should be required for publication, several specific analyses/comparisons that would help the manuscript are detailed below.

References:
The References are thorough and appropriate with several concerns expressed below.

Clarity and context:
The manuscript overall, including abstract, introduction, and conclusions are very well done, with the few concerns expressed below.
The authors skillfully contextualize their results within the broader landscape of ipi/nivo for advanced-stage melanoma.

In all, the manuscript is highly impactful and impressively executed; the intratumoral approach shown herein has immediate clinical relevance for vulnerable patient groups, and the authors outline a clear vision for future trials where this technique may minimize barriers and expand access to checkpoint blockade therapy. This work is a commendable step forward in personalizing immunotherapy and optimizing risk-benefit profiles for diverse cancer populations.

Reply to Referee #4:

We thank Referee #4 for his comprehensive assessment of our work, for underscoring all these good points about our results and for his favorable review of our manuscript.

Referee #4:

MAJOR CONCERNS

Regarding data inclusion, the manuscript is fairly rigorous, however, there are significant exclusions which would strengthen the data:

1) The authors currently provide thorough immune correlative data comparisons between Durable Clinical Benefit (DCB) pts and non-DCB, which is illuminating as to mechanisms of efficacy (though a bit confusing since we sometimes use the DCB acronym to mean 'double checkpoint blockade'), however the authors should also include (in either primary or supplementary Figures) immune correlative data comparisons between the intratumoral and intravenous cohorts, regardless of whether statistically significant differences were observed (noting that the study was not powered for these differences).

Reply to Referee #4:

In accordance with the same request of Referee #2, those IT vs IV comparisons have now been added into Extended Data Figure 7.

Referee #4:

2) Given prior data demonstrating the predictive power of genomic instability, e.g. somatic copy-number alterations being associated with protumoral inflammation as well as resistance to PD-1 blockade, [Danlos et al. Cancer Discov. 2023] it would be of high interest to know whether these associations did or did not occur in the current study. This is of particular importance because the Danlos study demonstrated that protumoral inflammation either correlated with or caused Treg infiltration, which the current study (with i.t. or i.v. CTLA4 blockade) has now flipped to be a predictor of favorable clinical outcome.

Reply to Referee #4:

We would like to thank Referee #4 for raising this important point. Indeed, we reported in 2023 that there was a higher number of somatic copy number alterations in the tumor whole exome of patients with No DCB and a positive correlation between the number of SCNA in the tumor and the circulating levels of IL6 in patients with advanced mesotheliomas treated with a combination of anti-PD1 and anti-angiogenic TKI. Here, in the context of first line advanced melanoma treated with anti-PD1 + anti-CTLA4 we did not find any predictive impact of the level of tumor SCNA on outcome and a modest correlation between the number of SCNA in tumors and the criculating levels of IL6.

Those new results have been added to the Extended Figure 3:

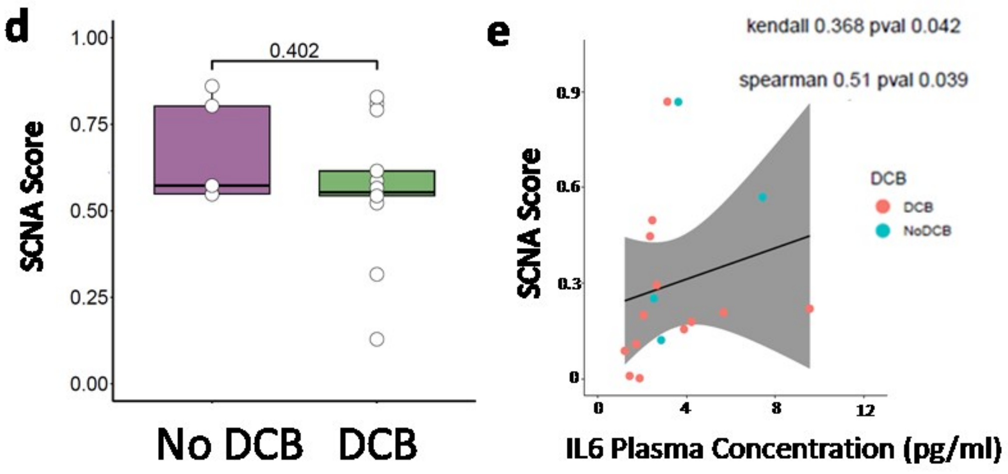

The legends and methods have been completed accordingly.

Referee #4:

3) The authors should be more precise and emphatic as to how this trial addresses another often-raised concerns regarding intratumoral therapy:
For example, regarding the concern whether the approach is sufficiently practical and such trials can be feasibly accrued/completed, the authors should clarify the total enrollment time per site (it appears that 2 sites were only opened for the latter part of the trial), and potentially put this in the context of a relevant comparator e.g. the MASTERKEY-265 trial which used intratumoral administration of oncolytic virus, accruing 700 patients at only 18 centers in 25 months.

Reply to Referee #4:

We thank Referee #4 to highlight this important point. We have now added the following sentence to our discussion to emphasize on the feasibility of IT procedures:

"Of note, as reported in the randomized phase III trial of intratumoral T-VEC + pembrolizumab (NCT02263508), intratumoral injection procedures did not impact our ability to enroll patients and accrual rates were similar to an intravenous only trial."

And have cited accordingly the Phase III T-VEC trial:

Chesney, J. A. et al. Randomized, Double-Blind, Placebo-Controlled, Global Phase III Trial of Talimogene Laherparepvec Combined With Pembrolizumab for Advanced Melanoma. J. Clin. Oncol. Off. J. Am. Soc. Clin. Oncol. 41, 528–540 (2023).

Referee #4:

Another example would be clearer data regarding the concern that the intratumoral approach may result in procedural (injection site) adverse events. The authors do indicate that there were no grade 3/4 procedural adverse events, however this data should also be provided for grade 1/2 events, presumably as an additional row in Extended Data Table 2.

Reply to Referee #4:

We agree with Referee #4 that we should underline the excellent tolerance of intratumoral ipilimumab, which had no drug specific adverse events during IT injections. Indeed, injections were performed with very thin (22G) needles and patients experienced no or limited (Grade 1/2) local pain upon IT injections.

Per request, we have added the incidence of Grade 1/2 procedure related pain to the Extended Data Table 2 and have added the following sentence to the Results section:

"Only localized grade 1-2 pain was experienced for some patients at the injection site (Extended Data Table 2). Overall, intratumoral ipilimumab was very well tolerated." [page 6; line 23]

Referee #4:

4) For the readership of Nature, the authors should present more clinical context in Introduction or Discussion for relevant comparators of nivo + i.t. ipi e.g. how did their results compare to the seminal trial of nivo + anti-LAG-3 [Tawbi HA et al, NEJM 2022] per safety and efficacy metrics (i.e. PFS).

Reply to Referee #4:

We agree with Referee #4 that our results should be discussed in the context of the anti-PD1 + anti-LAG3 data from the RELATIVITY-047 trial (initial results from Tawbi et al 2022 but most recent update with ORR and OS from Long et al 2023), and the recent results in RELATIVITY-048, demonstrated encouraging efficacy of the nivolumab + ipilimumab + relatlimab triplet combination, with a confirmed ORR of 58.7% and a 48-mo OS rate of 69.1% superior to doublet therapies.

We have added the following sentence to our discussion:

"Those results should also take into consideration the added value of anti-LAG3 antibodies when combined with anti-PD1 antibodies, and the promising superior activity of the triplet combination of anti-PD1, anti-LAG3 and anti-CTLA4 antibodies illustrating that intratumoral ipilimumab could also have an interest to maximize the intralesional exposure and dose dependent efficacy of anti-CTLA4 while mitigating the risk of irAEs for patients receiving a combination of anti-PD1 + anti-LAG3 IV." [page 15; line 29]

And cited the corresponding references:

- *V., L. G. et al. Overall Survival and Response with Nivolumab and Relatlimab in Advanced Melanoma. NEJM Evid. 2, EVIDoa2200239 (2023).*
- *Ascierto, P. A. et al. Efficacy and safety of triplet nivolumab, relatlimab, and ipilimumab (NIVO + RELA + IPI) in advanced melanoma: Results from RELATIVITY-048. J. Clin. Oncol. 42, 9504 (2024).*

Referee #4:

5) Again, for clinical context, the significance of studying nivo + i.t. ipi is only as great as the unmet need with the standard therapy (nivo + i.v. ipi). The manuscript would be strengthened by providing some data regarding what proportions of patients actually receive (nivo + i.v. ipi) in real world datasets e.g. those provided by [C T van Zeijl, M et al., J Immunother. 2023] or [Klein-Brill A et al., BJC Rep. 2024] or other sources.

Reply to Referee #4:

Indeed, real world implementation of IV ipilimumab+nivolumab is limited by the incidence of severe irAEs. We have added this sentence to our introduction with two real world data references (one US & one EU) supporting equivalent uptake of the combination therapy in practice.

"Such risk of severe irAEs limits the use of this combination therapy to ~50% of patients in real world setting despite its overall survival benefits." [page 4; line 13]

With the two references:

Billard K, et al. The efficacy and safety of first-line metastatic melanoma treatment with ipilimumab + nivolumab vs. nivolumab in a real-world setting. Br J Dermatol. 2025 May 19;192(6):1096-1105. doi: 10.1093/bjd/ljae470. PMID: 39605282.

Klein-Brill A, et al. Comparative efficacy of combined CTLA-4 and PD-1 blockade vs. PD-1 monotherapy in metastatic melanoma: a real-world study. BJC Rep. 2024 Feb 13;2(1):14. doi: 10.1038/s44276-024-00041-1. PMID: 39516682; PMCID: PMC11524072.

Referee #4:

MINOR CONCERNS
-The term "Biologic abnormalities" in Extended Data Table 2 is unclear and, particularly as there are 11 patients with Grade 3/4 "Biologic abnormalities" (between the two arms) this should explained in some granular detail.

Reply to Referee #4:

Biological abnormalities have now been added to the Extended Data Table 2.

Referee #4:

-Interleukin-2 is misspelled “Interleukine-2”

Reply to Referee #4:

We thank Referee #4 for his vigilance. Interleukin is now spelled correctly.

Referee #4:

-The authors do not seem to cite the most relevant study for clinical context, the 10-year update of Checkmate 067 – Wolchok J et al., N Engl J Med. 2025 (instead citing the Wolchok JCO 2022 update), this should be fixed. This is critical, as the latest (10-year) follow up indicates that there is still no survival benefit to intravenous nivo+ipi as compared to nivo alone. This is particularly important for the Nature readership (as opposed to Lancet or NEJM) that may not be aware that the ‘gold standard’ (intravenous) therapy is associated with high toxicity but not prolonged survival.

Reply to Referee #4:

We thank Referee#4 for this remark and have replaced the 2022 reference by the 2025 most recent update accordingly.
However we respectfully disagree with the interpretation of Referee#4 regarding the results of the Checkmate 067 trial. Indeed, this trial was designed to compare the ipilimumab+nivolumab and the nivolumab alone arms to the ipilimumab alone arm, but was not statistically powered to compare the ipilimumab+nivolumab arm vs the nivolumab arm. The overall survival and the melanoma-specific survival numbers were numerically higher in the ipilimumab+nivolumab combination arm than in the nivolumab monotherapy arm, although this effect seemed driven by some subgroups (notably BRAF mutated tumors, PD-L1 low tumors and LDH high patients).
We believe that our intra-patient comparison of ORR between injected vs non-injected lesions further illustrates the value of anti-CTLA4 therapy on top of anti-PD1 therapy.

Referee #4:

-The manuscript notes that a prior study of intratumoral ipilimumab [Ray et al Oncotarget . 2016] observed significant injection site reactions / ulcerations in 5 of 12 patients, though none were observed in this study. Given that large discrepancy, it would be helpful if the authors could present some hypothesis (or data) attempting to explain the difference.

Reply to Referee #4:

Indeed, the tumor ulcerations reported by Ray et al was a consequence of IL-2 rather than ipilimumab as we have not observed any ulcerations and intralesional IL2 alone induces tumor ulcerations.

As requested per Referee #4, we have added this comment to our Discussion:

"Therefore, the high incidence of ulcerations reported by Ray et al upon intralesional ipilimumab + IL-2 was likely due to IL-2 as supported by similar observations with intratumoral IL-2 monotherapy." [page 16; line 3]

And cited the supporting reference:

Radny P, et al. Phase II trial of intralesional therapy with interleukin-2 in soft-tissue melanoma metastases. Br J Cancer. 2003 Nov 3;89(9):1620-6. doi: 10.1038/sj.bjc.6601320. PMID: 14583759; PMCID: PMC2394422.

Referee #4:

-It is of some concern that the Fc Receptor data (Fig 6H-I) demonstrating improved clinical outcomes for patients whose tumors contain higher a priori levels of FCGR1A, FCGR3A, FCGR3B is obtained from transcriptomics, it would be strengthened by corroborative protein-level data if available.

Reply to Referee #4:

This observation was unexpected and therefore was not included in our flow cytometry panels performed on our prospectively collected fresh tumor biopsies. Unfortunately, we have run out of our FFPE material while performing our initial IHC stainings for (CD8, CD20, FOXP3,…) and therefore we don't have enough tumor material left to explore this question.

In order to address Referee #4 comment, we have looked for public datasets with paired RNA and protein expression. Overall, we found that there was a positive correlation between CD16 protein expression and its FCGR3A gene expression.

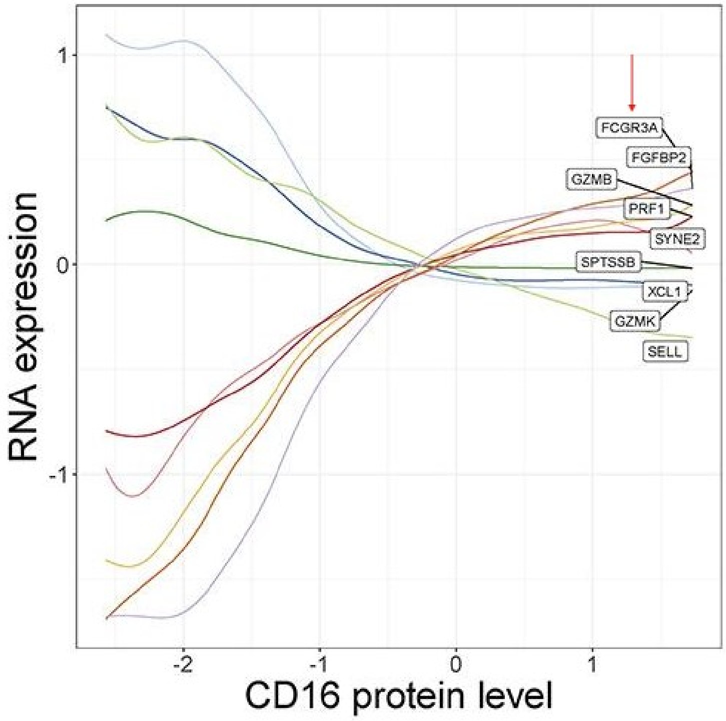

From Zhang B, et al Multimodal single-cell datasets characterize antigen-specific CD8+ T cells across SARS-CoV-2 vaccination and infection. Nat Immunol. 2023 Oct;24(10):1725-1734. doi: 10.1038/s41590-023-01608-9. Epub 2023 Sep 21. PMID: 37735591; PMCID: PMC10522491.

We believe this relevant question deserves a dedicated research project to describe at the protein level which subsets of cells are expressing membrane FcgRs, their potential interactions between them (e.g co-expression of activating and inhibitory FcgRs), and why primary refractory patients are not expressing those receptors in their TME.
